# Biomimetic liposomes in drug delivery: from design mechanisms to applications

**DOI:** 10.1039/d5cs00440c

**Published:** 2025-12-09

**Authors:** Zhi Li, Mengwen Li, Jianqin Lu

**Affiliations:** a Skaggs Pharmaceutical Sciences Center, Department of Pharmacology & Toxicology, R. Ken Coit College of Pharmacy, The University of Arizona Tucson AZ 85721 USA lu6@arizona.edu; b NCI-designated University of Arizona Comprehensive Cancer Center Tucson AZ 85721 USA; c BIO5 Institute, The University of Arizona Tucson AZ 85721 USA; d Southwest Environmental Health Sciences Center, The University of Arizona Tucson AZ 85721 USA

## Abstract

Recent advancements in nanocarriers, particularly liposomes, have shown promising prospects for enhancing the pharmacokinetics, biodistribution, and therapeutic efficacy of chemotherapeutic drugs. However, liposome-based drug delivery systems are often constrained by high immunogenicity, poor targeting efficiency, and limited functional capabilities. In this context, the exploration of biomimetic liposomes has revealed their potential in targeted therapy, immune camouflage, immune modulation, gene delivery and vaccine development. By integrating the beneficial features of functional molecules and natural cell membrane components with the unique properties of liposomes, biomimetic liposomes have demonstrated considerable promise in drug delivery. This review aims to emphasize recent progress in biomimetic liposomes and systematically elucidate their design mechanisms and preparation methods. Additionally, it provides a comprehensive overview of the current applications of biomimetic liposomes as an innovative drug delivery platform, with the goal of advancing knowledge for their effective utilization.

## Abstract 2

This review summarizes advances in biomimetic liposome-based delivery systems, highlighting design strategies such as membrane integration and applications in targeted therapy, imaging, immunomodulation, gene delivery, and vaccines.

## Graphical abstract

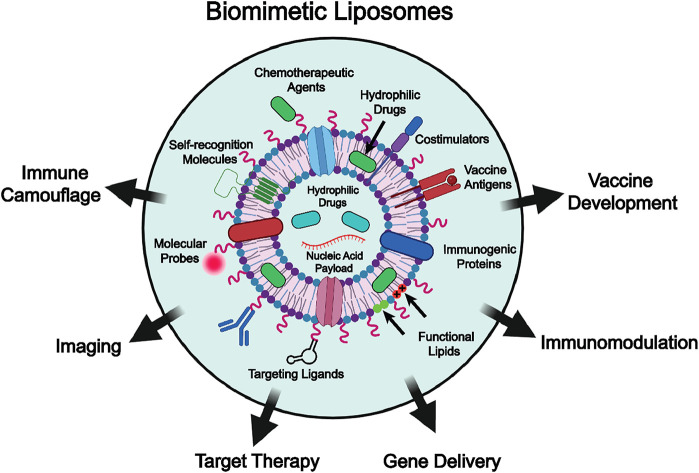

## Introduction

Targeted drug delivery denotes the transport of pharmaceutical agents to designated sites within the body through various strategies and technologies, intended to ensure optimal therapeutic outcomes.^1–4^ In traditional drug delivery, drugs are administered through established methods such as oral intake and injection, which deliver drugs into the systemic circulation rather than directly to specific target sites.^5^ The rapid development of nanotechnology has brought about a revolution in the field of drug delivery.^6^ Numerous studies have begun to focus on nanoscale drug carriers, such as polymeric vesicles/micelles, inorganic nanosystems, and liposomes.^6–9^ These carriers can precisely deliver drugs to targeted lesions or sites within the body, significantly enhancing the therapeutic effects of the drugs and reducing damage to healthy tissues. Among various nanocarriers, liposomes have shown outstanding performance, resulting in several drug-loaded liposomes being approved by the US Food and Drug Administration (FDA).^10,11^ For example, Doxil, the first FDA-approved liposomal drug, demonstrated an extended circulation time in the bloodstream, reduced toxicity, and increased drug accumulation at tumor sites compared to its free form, doxorubicin.^12^ A milestone event was the use of lipid nanoparticles (LNPs) with a liposome-like architecture, which evolved from conventional liposomes through the incorporation of ionizable lipids to enable efficient nucleic acid encapsulation and delivery, as carriers for mRNA COVID-19 vaccines during the SARS-CoV-2 pandemic, ultimately saving millions of lives.^13–15^ This provides strong evidence for the safety and effectiveness of functional liposomes as nanocarriers and promotes their rapid progress in drug delivery applications.

Liposomes are small, spherical vesicles composed of one or more phospholipid bilayers enclosing an aqueous core.^16,17^ They can encapsulate a diverse range of therapeutic agents, including small molecules, peptides, proteins, and nucleic acids. Many conventional drugs undergo chemical modification to enhance their lipophilicity, which improves their encapsulation efficiency and retention within the liposome and increases the overall formulation stability.^18,19^ Such modifications not only facilitate effective drug loading but also help maintain therapeutic concentrations over extended periods, conferring controlled drug release and improving pharmacokinetic profiles.^20^ These strategies demonstrate the versatility of liposomes as a platform for diverse therapeutic modalities, from conventional small molecules to more complex biologics. Liposomes can not only protect their cargo from degradation in the bloodstream, enhancing pharmacokinetics and therapeutic efficacy, but also reduce the systemic toxicity.^21^ Moreover, the enhanced permeability and retention (EPR) effect enables liposomes to preferentially accumulate at tumor sites, which leads to increased drug release and deeper tumor penetration.^22,23^ Consequently, most FDA-approved cancer nanomedicines are liposome-based formulations. However, these liposomal vesicles face several biological barriers that limit their optimal biodistribution and therapeutic efficacy. A primary challenge is that, as foreign entities, liposomes are readily recognized and cleared by the mononuclear phagocyte system (MPS).^24–26^ Moreover, the lack of intrinsic active targeting mechanisms often results in off-target accumulation, potentially causing adverse effects on healthy tissues and cells. Additionally, traditional liposomes have been used exclusively as drug carriers and lack multifunctionality, which further limits their development and clinical translation.

**Fig. 1:**
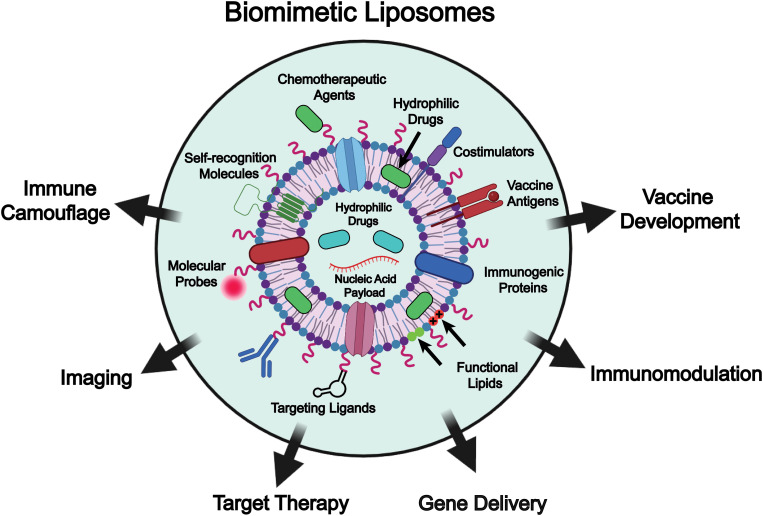
Schematic illustration of the biomimetic liposomes combines functional molecules (*e.g.*, targeting ligands, molecular probes, chemotherapeutic agents, and ionizable lipids) or naturally derived components (*e.g.*, immunogenic proteins, antigens, costimulatory molecules, and self-recognition molecules) on their membrane, as well as their applications in drug delivery, including immune camouflage, imaging, target therapy, gene delivery, immunomodulation, and vaccine development. Image created with BioRender.com.

To address these challenges, various functional molecules, such as self-recognition moieties, targeting ligands, molecular probes, and ionizable lipids, as well as naturally derived components including immunogenic proteins, vaccine antigens, and costimulatory molecules, are incorporated into liposome-based drug delivery systems to enable biomimetic engineering of the liposomes (Fig. 1).^27–34^ These bioinspired components not only confer immune camouflage or special targeting capabilities to liposomes through interactions between cells and tissues, but also enrich the functionality (*e.g.*, gene delivery, immunomodulation, and vaccine development) of the liposomes, potentiating the efficacy of chemotherapy. Functionalization of liposomes through membrane fusion with biological membranes, such as those derived from macrophages and exosomes, endows them with immune evasion, targeted binding, and lysosomal escape properties, and ultimately permits precise therapeutic delivery to pathological sites.^35^ In addition, these membrane-derived components can modulate cytokine expression and normalize the immune microenvironment, synergistically enhancing the efficacy of chemotherapy.^36^ Moreover, liposomes integrated with pathogen membrane proteins or fused with outer membrane vesicles (OMVs) can mimic pathogen-associated molecular pattern (PAMP)-like presentations, enabling precise targeting of antigen-presenting cells and eliciting robust immune responses, thereby demonstrating great potential for clinical applications.^37–39^ In addition, the incorporation of certain bacterial membrane components, such as c-type cytochrome-based electron channels, into liposomal membranes can simulate extracellular electron transfer to surrounding oxygen, which markedly enhances the generation of superoxide anions under low-dose (1 Gy) X-ray irradiation and consequently leads to improved efficacy of radio-dynamic therapy.^38^ Taken together, biomimetic liposomes represent a significant advancement in drug delivery systems. They not only improve the immunogenicity, targeting specificity, and therapeutic efficacy of liposomal formulations but also provide the liposomal platform with enhanced functional diversity, which expands their potential applications in precision medicine.

Although biomimetic liposomes have garnered increasing attention in drug delivery and the broader biomedical engineering field, comprehensive reviews systematically addressing their design, preparation, and applications remain limited.^2,40–55^ In this review, we summarize recent advances in the formulation of biomimetic liposome-based drug delivery systems, focusing on the design mechanisms (*e.g.*, liposome architecture, surface modification, and integration of natural membrane components), and preparation strategies (*e.g.*, “top-down” and “bottom-up” approaches). Furthermore, we highlight diverse applications of biomimetic liposomes in drug delivery, including immune camouflage, targeted therapy, immune modulation, gene delivery and vaccine development (Fig. 1). It is important to note that the terms biomimetic liposomes and functionalized liposomes are occasionally used interchangeably in the literature, which can create conceptual ambiguity. In this review, we delineate the two while recognizing their conceptual overlap. Specifically, biomimetic liposomes are described as systems designed to emulate natural biological structures or interactions, for example through the incorporation of different lipid species or natural cell membrane components to facilitate cell–lipid recognition. Functionalized liposomes, in contrast, are engineered with additional moieties such as lipopeptides, PEG, antibodies, or enzymes to achieve properties like targeting, long circulation, or stimulus-responsiveness. Conceptually, however, many functionalized designs can also be regarded as biomimetic, since they recapitulate strategies that cells naturally employ for recognition, uptake, and immune evasion. To maintain consistency, we have primarily adopted the term biomimetic liposomes in this review, while noting that certain functionalized liposomes may also fall within this broader biomimetic framework. Finally, we provide our perspectives on the current challenges in the engineering of biomimetic liposomes, including issues related to large-scale production and quality control, discuss potential solutions, and highlight future directions for their development and application (Table 1).

**Table 1:** Summary of major findings and representative studies categorized by liposome designs and mechanisms, preparation methods, applications, and summary and outlook. The table highlights the key strategies, corresponding mechanisms, and outcomes reported in recent studies, providing a comparative overview of current advancements in biomimetic liposome research

Section	Strategy	Major findings	Ref.
Design and mechanisms	The selection of lipids	Natural or synthetic lipids offer reactive sites for versatile biomimetic modifications	78, 79 and 156
		Designing diverse lipid structures enables targeted delivery and immune camouflage	
	Surface modifications	Covalent lipid–drug conjugation improves bioavailability and therapeutic efficacy	9 and 36
		Reversible hydrophobic interactions preserve protein integrity in biomimetic liposomes	
	Membrane resources	Macrophage membrane-integrated biomimetic liposomes hold promise for the treatment of inflammatory diseases	35 and 38
		Bacterial membrane components enhance the X-ray photodynamic efficacy of biomimetic liposomes	
Preparation methods	Membrane incorporation	Freeze–thaw natural cell membranes and extrude into stable biomimetic liposomes	100, 136 and 138
		“Bottom-up” biomimetic liposomes better preserve membrane protein integrity and function	
	Membrane fusion	Membrane fusion of EVs with liposomes enhances stability, targeting, and drug loading	73
Applications	Targeted therapy	Antibody-modified biomimetic liposomes enable *in vivo* activation of CAR-T therapy	152 and 153
		XMV-fused biomimetic liposome vaccines elicit strong antitumor immune responses	
	Immune camouflage	Polymer-locked fusogenic liposomes enable BBB translocation for targeted brain drug delivery	154 and 156
		Evans blue-modified biomimetic lipid nanoparticles efficiently target lymph nodes and evade immune clearance	
	Imaging	Biomimetic liposomes with disease-targeting and immune-evasive properties have emerged as promising platforms for biomedical imaging	88 and 161
	Immunomodulation	Membrane-integrated biomimetic liposomes mimic natural cell interactions, combining immune evasion and targeting, and hold promise for immune regulation and inflammation therapy	116 and 163
	Gene delivery	Siloxane-based ionizable lipidoids and SiLNPs improve mRNA stability and enable organ-specific *in vivo* delivery	70
	Vaccine development	Biomimetic erythrocyte membrane-liposomes enable spleen-targeted delivery of iPSC proteins, inducing tumor-specific immunity and suppressing tumor progression	153 and 167
Summary and outlook	Challenges and potential solutions	Several key challenges (*e.g.*, stability and long-term storage issues) should be addressed to advance the clinical translation of biomimetic liposomes	20, 136 and 137
	Production at industrial scale	Some issues, such as raw materials and reproducibility, as well as manufacturing techniques and scale-up, need to be carefully considered before industrial production	66, 170 and 171

## Design and mechanisms

**Fig. 2:**
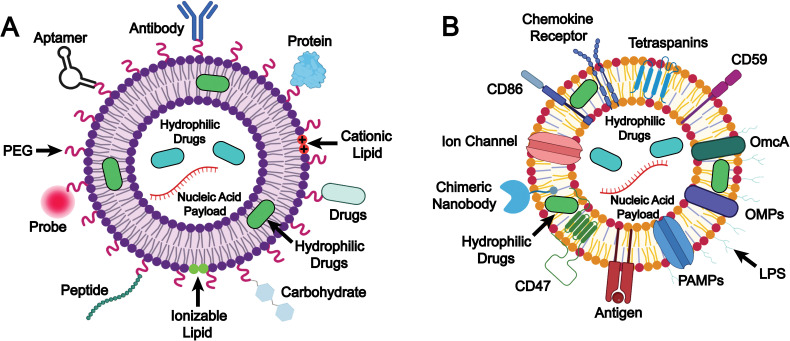
Schematic illustration of the design and mechanisms of biomimetic liposomes through (A) the selection of lipids and surface modifications, and (B) the integration of natural membrane components. Image created with BioRender.com.

Biomimetic liposomes constitute an advanced strategy in liposome-based drug delivery through the combination of the structural benefits of conventional liposomes and the features that mimic natural intercellular interactions. This design enhances immune camouflage, targeting specificity, and functional versatility. The underlying design and mechanisms play a crucial role in drug delivery performance, as they enhance drug stability and pharmacokinetics, increase targeting efficiency, reduce off-target effects, and achieve synergistic therapeutic outcomes. In this chapter, we focus on two key strategies for formulating biomimetic liposomes: (1) the selection of lipids and surface modifications, and (2) the integration of natural membrane components (Fig. 2).

### The selection of lipids and surface modifications

The selection of specific lipids combined with strategic surface modifications represents a fundamental approach in the design of biomimetic liposomes (Fig. 2A).

#### Selection of lipids

Lipids, particularly phospholipids, are an essential core component in the structural and functional design of liposomes. Phosphatidylcholines (PCs), including 1,2-distearoyl-*sn-glycero*-3-phosphocholine (DSPC), egg phosphatidylcholine (EPC), and 1,2-dioleoyl-*sn-glycero*-3-phosphocholine (DOPC), are widely used in FDA-approved liposomal formulations (*e.g.*, Doxil, Myocet, and DepoDur) due to their biocompatibility and capacity to form stable liposomes.^56–62^ Beyond PCs, other phospholipids such as phosphatidylglycerols (PGs), sphingomyelin (SM), and phosphatidylethanolamines (PEs) are also commonly employed in FDA-approved liposomal formulations.^63–65^ Due to its ability to improve membrane stability and prolong systemic circulation, SM is used in the FDA-approved liposomal vincristine formulation Marqibo.^66,67^ Different types of lipid molecules not only contribute to structural variations in liposomes but also significantly influence their physicochemical properties and biological functions. For example, to achieve more efficient loading and precise delivery of nucleic acid-based therapeutics, particularly mRNA, into target organs or cells, cationic lipids or ionizable lipid molecules are commonly employed in the preparation of liposomal formulations.^68–70^ The COVID-19 mRNA vaccine developed by Moderna utilized an ionizable lipid molecule, SM-102, to encapsulate the SARS-CoV-2 spike protein-encoding mRNA.^71^

Lipids can be classified into natural and synthetic types based on their source. Natural lipids, such as PC, PE, and phosphatidylserine (PS), are mainly components of cell membranes or organelle membranes, and often exhibit high biocompatibility, low immunogenicity, and good biodegradability.^72,73^ Given these advantages, many FDA-approved liposomal formulations utilize natural lipids. For example, Ambisome, which is a liposomal formulation of amphotericin B approved by the FDA for the treatment of systemic fungal infections, employs hydrogenated soy phosphatidylcholine (HSPC), a natural phospholipid, to form a lipid bilayer and enhance membrane stability and biocompatibility.^74,75^ Since most natural lipid molecules are ubiquitously present within cells, liposomes composed of natural lipids generally lack inherent targeting capability. However, in some cases, natural lipids can still contribute to targeted delivery due to their species-specific interactions with the biological system. For instance, PS is typically exposed on the surface of damaged or apoptotic cell membranes, serving as an “eat-me” signal that promotes recognition and phagocytosis by macrophages.^76,77^ This specific interaction with immune cells provides a basis for targeted drug delivery to inflammatory sites, where macrophages are highly accumulated.

**Fig. 3:**
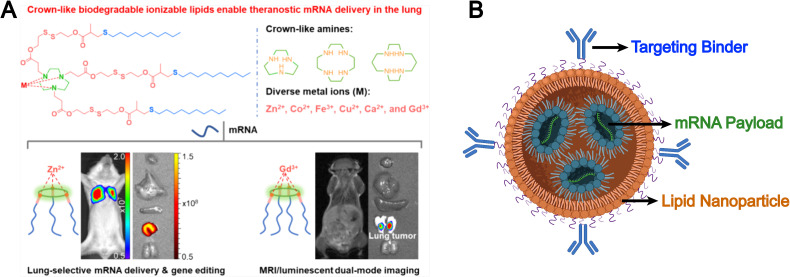
(A) Chemical structures of corona-shaped biodegradable ionizable lipid, and their application in targeted mRNA delivery/imaging to the lungs. Reproduced from ref. 78 with permission from the American Chemical Society, copyright (2024). (B) Schematic representation of a targeted lipid nanoparticle. Image adapted from ref. 79, Hunter *et al.*, 2025 and created with BioRender.com.

Synthetic lipids are another source for liposomal formulations. Unlike natural lipids, synthetic lipids offer a high degree of structural tunability, allowing for the rational incorporation of advanced functionalities, such as molecular targeting, stimuli responsiveness, and immune modulation, into liposomal platforms. For example, Chen *et al.* addressed the limitation of conventional liposomes, which tend to preferentially accumulate in the liver and exhibit poor delivery efficiency to other organs such as the lungs. They developed a novel corona-shaped biodegradable ionizable lipid that achieves selective delivery to the lungs through metal coordination chemistry (Fig. 3A).^78^ Moreover, synthetic lipids possess diverse chemically reactive moieties within their head groups, enabling extensive functionalization and tailored modification of liposomal systems. For example, Hunter *et al.* employed maleimide-thiol chemistry to conjugate CD8 antibodies onto the surface of liposomes composed of 1,2-distearoyl-*sn-glycero*-3-phosphoethanolamine-*N*-[maleimide(polyethylene glycol)] (DSPE-PEG-Mal), effectively evading hepatic clearance and enabling precise targeting of CD8^+^ T cells, which accumulate in immune organs such as the spleen (Fig. 3B).^79^ The liposomal system subsequently released mRNA encoding CD19-specific CARs, thereby generating functional CD19-targeted CAR-T cells *in vivo*. This study demonstrates that synthetic lipid systems can also be expanded for applications in precision drug delivery and cell therapy through biomimetic design (*e.g.*, mimicking immune recognition and regulation). However, they inevitably face challenges such as high immunogenicity, biosafety, and production costs.

#### Surface modifications

Biomimetic surface functionalization offers an effective strategy to improve the biocompatibility, biodistribution, and targeting efficiency of liposomal systems. By introducing such surface modifications, liposomes acquire biomimetic properties that enhance drug delivery performance in biological environments. Depending on the binding mechanism, surface modification strategies can be broadly categorized into covalent and non-covalent approaches.

Covalent modification entails attaching functional molecules to the surface of liposomes through the formation of stable covalent bonds.^80^ A critical step in this process is the introduction of chemically active sites on the liposomal surface. The most used lipid molecule is DSPE-PEG, which contains various terminal active sites and can self-assemble onto the liposome surface during formation, exposing these sites on the outer membrane.^81–83^ These exposed sites can then undergo simple chemical conjugation reactions (*e.g.*, click chemistry) with functional molecules bearing active groups such as thiols or amines. The incorporation of such functional molecules enhances interactions between the liposomes and specific target cells *via* ligand–receptor recognition, thereby improving the efficiency of drug or gene delivery. For instance, Kim *et al.* employed click chemistry to conjugate the macrophage-targeting peptide CRV onto the surface of liposomes. This modification enabled selective recognition of macrophages and facilitated the direct delivery of oligonucleotide payloads into the cytoplasm, effectively bypassing the endocytosis pathway.^84^

**Fig. 4:**
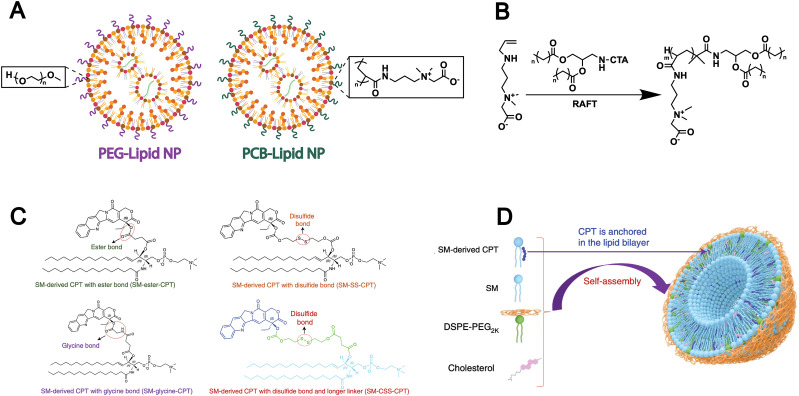
(A) Schematic of this work, where PEG-lipid was fully replaced by PCB-lipid in the LNP-containing ionizable cationic lipid, phospholipid, cholesterol and mRNA cargo. (B) Reversible addition–fragmentation chain transfer polymerization (RAFT) was used to synthesize different variants of PCB-lipids with different average carboxybetaine repeat units (*m* = ∼5, *m* = ∼15 or *m* = ∼27) and different acyl chain lengths (*n* = 12 or *n* = 16). Adapted from ref. 86 and created with BioRender.com. (C) Conjugation of SM and CPT resulted in SM-derived CPTs with either an ester bond (SM-ester-CPT), a disulfide linkage (SM-SS-CPT), a glycine bond (SM-glycine-CPT), or a disulfide linkage and a longer linker (SM-CSS-CPT). (D) Schematic depicting the self-assembly of SM-CPT into a camptothesome. Reproduced from ref. 9 with permission from Springer Nature, copyright (2021).

However, the extensive application of PEG in drug delivery systems has been shown to elicit the formation of anti-PEG antibodies, which can accelerate systemic clearance and compromise therapeutic efficacy.^85,86^ To address this issue, researchers are developing non-PEG modification strategies by grafting alternative hydrophilic polymers onto lipids to replace PEG. For example, Luozhong *et al.* modified lipids with a novel PEG alternative-poly(carboxybetaine) (PCB) and subsequently assembled them into liposomes for mRNA delivery (Fig. 4A).^86^ PCB-lipids were synthesized *via* RAFT polymerization using a “graft-from” approach. The lipid library included variants with poly(carboxybetaine) chains of 2, 4, or 7 kDa and two distinct acyl chains: DMG (1,2-dimyristoyl-rac-*glycero*-) and DSG (1,2-distearoyl-*rac-glycero*-) (Fig. 4B). The resulting PCB-functionalized lipids were formulated into an mRNA delivery system, which demonstrated markedly enhanced transfection efficiency and effectively mitigated the accelerated blood clearance effect commonly associated with PEGylated lipid nanoparticles. This PEG-alternative strategy conforms to biomimetic design principles, as it mimics natural cell-surface properties and allows immune evasion and prolonged circulation, which highlights its potential as a biomimetic approach in lipid-based drug delivery.

In addition to synthetic lipids bearing chemically reactive groups, some naturally derived lipid molecules (*e.g.*, SM) also contain functional groups such as hydroxyl and carboxyl groups, offering opportunities for surface modification of liposomes formed from natural lipids. For example, given the unique features of the tumour microenvironment, Wang *et al.* designed three different linkages-ester, disulfide, and thioketal bonds to connect the SM-camptothecin (SM-CPT) conjugates (Fig. 4C).^9^ Each linkage is specifically responsive to a particular stimulus that exists at high levels in tumour sites (hydrolases, glutathione (GSH), and reactive oxygen species (ROS), respectively), thereby enabling the on-demand release of CPT. Then, they successfully generated a SM-derived CPT nanotherapeutic vesicle platform (camptothesomes) through self-assembly (Fig. 4D). The covalent conjugation of lipid molecules with chemotherapeutic agents has been shown to significantly enhance drug bioavailability and tumor accumulation, thereby improving antitumor efficacy. These are attributed to the modifiable nature of natural lipids such as SM, which enables responsive drug release and supports the design of biomimetic liposomes with improved targeting and biocompatibility.

**Fig. 5:**
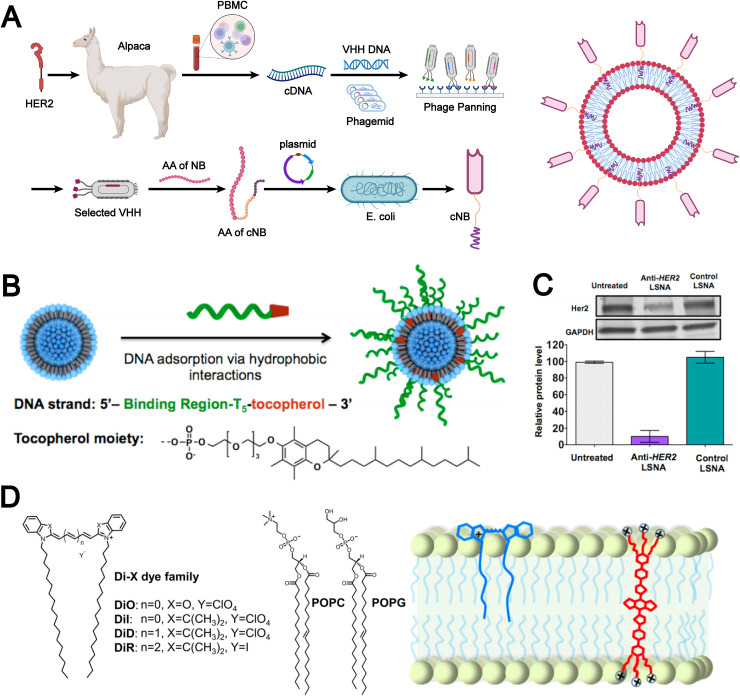
(A) Flowchart illustrating the manufacturing processes of chimeric nanobody (cNB) and its liposomal platform (cNB-LP). Adapted from ref. 36and created with BioRender.com. (B) Assembly of liposomal spherical nucleic acids (SNAs) from DOPC small unilamellar vesicles (SUVs) and tocopherol-modified DNA. (C) Human epidermal growth factor receptor 2 (HER2) gene knockdown in SKOV-3 cells using anti-HER2 liposomal SNAs. Reproduced from ref. 87 with permission from the American Chemical Society, copyright (2014). (D) Chemical structures of the Di family of carbocyanine dyes alongside representative phospholipids, neutral phospholipid 1-palmitoyl-2-oleoyl-*glycero*-3-phosphocholine (POPC) and negatively charged 1-palmitoyl-2-oleoyl-*sn-glycero*-3-phospho-(1′-rac-glycerol) sodium (POPG) (left). Schematic illustration showcasing the binding of DiR and conjugated electrolytes (CE) to the lipid membrane (right). Reproduced from ref. 88 with permission from Wiley-VCH, copyright (2024).

Non-covalent modification employs reversible molecular forces, such as hydrogen bonds, electrostatic attractions, π–π stacking, and hydrophobic effects, to functionalize the liposomal surface with bioactive molecules, thereby enhancing the biomimetic properties of liposomes. For example, Rahman *et al.* reported a chemical modification-free biophysical strategy for constructing immunoliposomes in a single step *via* the self-assembly of chimeric nanobodies (cNBs) into the liposomal bilayer (Fig. 5A).^36^ The cNBs, composed of a nanobody targeting human epidermal growth factor receptor 2 (HER2), a flexible peptide linker, and a hydrophobic single transmembrane domain, can be efficiently anchored onto 100-nm sized liposomes without steric hindrance. This biomimetic immunoliposome, generated through non-covalent modification, markedly enhances the cytotoxicity of the encapsulated drug against HER2-overexpressing cancer cells and prolongs survival in cancer models. The absence of chemical modifications preserves native protein structure and function, which provides substantial potential for the development of precise and effective liposomal therapeutics.

In addition, Banga *et al.* designed DNA strands functionalized with a tocopherol tail, which were inserted into the lipid bilayer. This approach not only imparted biomimetic characteristics to the liposomes but also enhanced their structural stability (Fig. 5B).^87^ The resulting liposomal spherical nucleic acids (SNAs) exhibited promising potential for cellular transfection and gene regulation. Notably, anti-HER2 liposomal SNAs significantly downregulated HER2 protein expression levels (Fig. 5C). These findings highlight that hydrophobic interactions, as a common type of non-covalent force, play a vital role in the fabrication and modification of biomimetic liposomes.

Moreover, the molecular backbone structure plays a crucial role in determining the efficiency of hydrophobic interactions and lipid bilayer incorporation. Meng *et al.* designed three novel conjugated electrolyte (CE) near-infrared II fluorescent probes featuring twisted backbone conformations.^88^ During liposome preparation, CE molecules were incorporated into the lipid bilayer *via* a “passive” approach based on hydrophobic interactions. This strategy significantly enhanced membrane integration efficiency, achieving nearly 100% incorporation (Fig. 5D). Non-covalent modification imparts biomimetic liposomes with enhanced biocompatibility and functional flexibility through mild and reversible interactions, while preserving the native conformation of bioactive molecules. However, limitations in stability and controllability still restrict their performance in complex *in vivo* environments and large-scale manufacturing.

### Integration of natural membrane components

Integration of natural membrane components represents an advanced functionalization strategy, wherein natural membrane-derived elements, such as lipids and membrane proteins, are incorporated onto the surface of liposomes (Fig. 2B). This method preserves key structural and biological characteristics of the native membrane, either partially or entirely, thereby enhancing the biomimetic nature of the membrane-inspired liposomes. By recapitulating the architecture and functionality of cellular membranes, this approach significantly improves liposomal biocompatibility, target specificity, and physicochemical stability. Moreover, it enables the introduction of specialized functions, including immune modulation and vaccine development. Owing to these multifaceted advantages, biomimetic liposomes have emerged as a highly versatile platform for precision drug delivery, immunotherapy, and next-generation vaccine development.

#### Cell membrane resources

Cell membrane resources are crucial for the formulation of biomimetic liposomes through the integration of natural membrane components. Different cell membranes significantly affect the stability and functionality of biomimetic liposomes.

Among the earliest membrane resources employed for liposomal functionalization were red blood cell membranes and tumor cell membranes. Liposomes incorporating red blood cell membrane components benefit from the presence of “self-recognition” markers such as CD47 glycoprotein and CD59 (protectin), which enable effective evasion of macrophage-mediated clearance.^89–91^ This results in prolonged circulation time in the bloodstream and enhanced passive accumulation of therapeutics in target tissues or cells. In contrast, tumor cell membranes contain a diverse array of specific biological constituents that confer unique biomedical functionalities to liposomes during biomimetic modification. Notably, tumor-associated antigens (TAAs) derived from tumor cell membranes can improve the tumor-targeting ability of biomimetic liposomes *via* antigen–receptor interactions.^92–94^ Moreover, TAAs serve as key components in the development of tumor nanovaccines, wherein TAA-loaded nanovaccines are delivered to antigen-presenting cells (APCs) *in vivo*, thereby eliciting a potent and specific anti-tumor T-cell immune response.

**Fig. 6:**
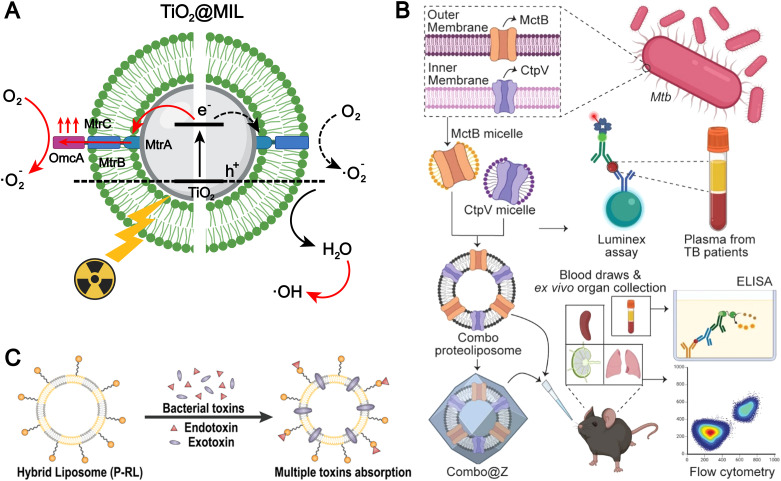
(A) The illustrated mechanisms of the X-ray-induced EET biomimicking for electron transport from the TiO_2_ core across the MtrCAB–OmcA-based electron channel to the surrounding oxygen for O_2_˙^−^. Adapted from ref. 38 and created with BioRender.com. (B) Utilization of membrane proteins as tuberculosis (TB) antigens for the development of a biomimetic liposomal vaccine. Reproduced from ref. 37 with permission from the American Chemical Society, copyright (2024). (C) Schematic illustration of P-RL. P-RL is fabricated by the fusion of polymyxin B-modified lipids and the red blood cell membrane. Benefiting from the two materials, the P-RL is capable of simultaneously absorbing endotoxins and exotoxins that are secreted by *E. coli*. Reproduced from ref. 95 with permission from the American Chemical Society, copyright (2021).

The bacterial membrane surface harbors numerous components that can be utilized for the fabrication of biomimetic liposomes.^40,95,96^ Chen *et al.* integrated the membrane protein complex MtrCAB–OmcA from Shewanella oneidensis MR-1 onto the surface of TiO_2_ liposome (TiO_2_@MIL) membranes to construct a liposome-based photocatalytic system mimicking extracellular electron transfer (EET) (Fig. 6A).^38^ Under X-ray irradiation, the MtrCAB–OmcA-mediated electron channel facilitates the electron transfer from the conduction band of excited TiO_2_ to oxygen, suppressing electron–hole recombination. This process enhances the generation of superoxide radicals (O_2_˙^−^) and indirectly promotes the formation of hydroxyl radicals (˙OH). Liposomal formulations modified with bacterial membrane components are more readily internalized *via* endocytic pathways, primarily due to the presence of hydrophobic and charged domains in bacterial membrane proteins, as well as pathogen-associated molecular patterns (PAMPs), which enhance the affinity between the liposomes and the cell membranes, thereby improving cellular uptake efficiency.

PAMPs can also activate pattern recognition receptors (PRRs) and induce an immune response. Therefore, incorporating these pathogen membrane proteins into liposomes to mimic the presentation in the pathogen is of great significance in the field of infectious disease vaccines. For example, Kumari *et al.* were the first to report the integration of two different transmembrane proteins, Cation Transporter Protein V (CtpV) and Mycobacterial Copper Transporter Protein B (MctB) which are from the *Mycobacterium tuberculosis*, into liposomes to formulate proteoliposomes that mimic PAMP-like presentation for the development of a novel tuberculosis (TB) vaccine (Fig. 6C).^37^ This liposome-based vaccines with mimic PAMP-like presentation generate robust immunological responses, which hold significant potential for clinical applications.

Bacteria membrane-inspired liposomes exhibit significant potential for diverse biomedical applications. However, bacterial membrane components may inherently carry toxic substances, including endotoxins and exotoxins, which pose the risk of inducing systemic inflammatory responses and immunotoxicity. Although red blood cells (RBCs) lack immune signaling functions, certain exotoxins can directly compromise RBC membrane integrity, leading to hemolysis. Furthermore, systemic inflammatory processes can exacerbate RBC injury *via* secondary mechanisms such as complement activation and oxidative stress. Drawing inspiration from this concept, Jiang *et al.* developed a polymyxin B (PMB)-modified RBC-mimetic hybrid liposome (P-RL).^95^ This nanosystem was succinctly fabricated by fusing PMB-functionalized lipids with RBC membranes to form an integrated hybrid membrane. Leveraging the strong affinity between PMB and *Escherichia coli* membranes, P-RL specifically adheres to and anchors on the bacterial surface (Fig. 6C). Moreover, the synergistic effect of the fused RBC membrane and PMB modification enables efficient neutralization of both endotoxins and exotoxins originating from the bacterial toxin sources. This RBC-mimetic hybrid liposome, integrated with antimicrobial peptide modification, achieves specific bacterial adhesion and dual neutralization of endotoxins and exotoxins, highlighting the unique advantages of biomimetic design in infection therapy. Nevertheless, the complexity of its composition and potential immunological risks (*e.g.*, variability of RBC sources and PMB-associated toxicity) may hinder clinical translation and large-scale application.

**Fig. 7:**
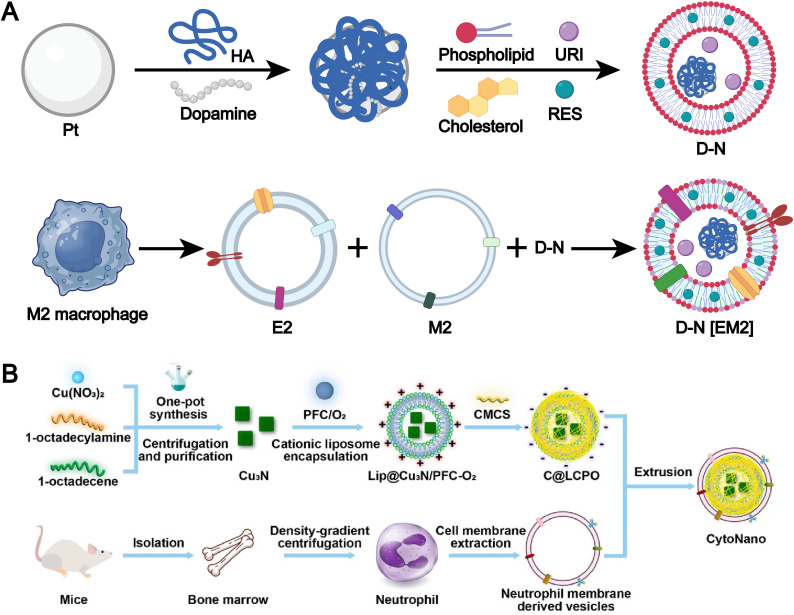
(A) Synthesis pathway of PtHD, D-N and D-N[EM2]. Adapted from ref. 35 and created with BioRender.com. (B) Design and synthesis of CytoNano. This elegantly engineered composite features a cationic liposome loaded with copper-nitride nanoparticles and oxygen-rich perfluorocarbon (Lip@Cu_3_N/PFC-O_2_), all wrapped in a sophisticated coating of neutrophil membrane and acid-responsive carboxymethylcellulose (CMCS, with an isoelectric point of 6.5). Its fabrication is accomplished through a detailed, integrative self-assembly process, meticulously combining these elements to target cancer cells precisely and effectively. Reproduced from ref. 109 with permission from the American Chemical Society, copyright (2024).

The membranes of immune cells, such as T cells, B cells, macrophages, and dendritic cells, have emerged as a valuable source for the fabrication of biomimetic liposomes.^47,97–102^ Immune cell membranes are intrinsically enriched with chemokine receptors, adhesion molecules, cytokine receptors, and costimulatory molecules, which collectively endow immune cell membrane-biomimetic liposomal systems with significantly enhanced targeting specificity and superior immunomodulatory capabilities. For example, Xu *et al.* developed a smart biomimetic nanosystem based on macrophage membranes and liposomes (Fig. 7A).^35^ First, uricase, platinum-in-hyaluronan/polydopamine nanozyme, and resveratrol were encapsulated within liposomes, which were then cloaked with a hybrid membrane derived from the fusion of M2 macrophage membranes and exosomes to form the smart biomimetic nanosystem. This drug delivery platform precisely targets inflamed joints, induces polarization of pro-inflammatory M1 macrophages, and promotes the local accumulation of anti-inflammatory M2 macrophages. Meanwhile, the synergistic action of uricase and nanozyme effectively reduces urate levels within the joints. This system represents a highly effective and minimally immunogenic strategy for multimodal gouty arthritis therapy.

Emerging studies also highlight the potential of macrophage membrane-integrated nanocarriers for targeted drug delivery to inflammatory sites.^47,98,103–107^ The inflammation-homing and therapeutic properties of these biomimetic systems are primarily attributed to the abundant expression of chemokine receptors, such as chemokine receptor type (CCR) 2 and CCR5, on the macrophage membrane, which enable recognition of chemokines (*e.g.*, CCL2, CCL5) secreted within the inflamed microenvironment.^108^ Additionally, the presence of integrins, selectins, and other adhesion molecules facilitates the active migration of liposomal carriers toward inflamed tissues and enhances their adhesion to vascular endothelium, thereby improving local accumulation and deep tissue penetration. Notably, M2-type macrophage membranes further contribute to therapeutic outcomes by modulating the immune microenvironment. They are capable of adsorbing pro-inflammatory cytokines including TNF-α, IL-6, and IFN-γ, which helps attenuate excessive inflammation and reduce oxidative stress through the downregulation of local ROS levels. Furthermore, these membranes are enriched with anti-inflammatory components such as IL-10 and TGF-β receptors, which can activate downstream signaling cascades (*e.g.*, IL-10/STAT3 and TGF-β/STAT6 pathways), ultimately facilitating the phenotypic reprogramming of M1 macrophages toward an anti-inflammatory M2-like state. Macrophage membrane-coated nanocarriers leverage biomimetic chemokine receptors and adhesion molecules for precise delivery to inflamed tissues, while M2-derived components offer unique benefits in immune modulation.

Neutrophils, like macrophages, exhibit potent inflammation-associated chemotaxis, allowing them to efficiently home to tumor microenvironments. For example, Li *et al.* developed a dual-responsive therapeutic system, termed “CytoNano,” by integrating a cationic liposome encapsulating copper nitride nanoparticles and oxygen-rich perfluorocarbon (Lip@Cu_3_N/PFC-O_2_) with a neutrophil membrane and acid-responsive carboxymethylcellulose (Fig. 7B).^109^ This system leverages the biomimetic nature of neutrophils and their responsiveness to acidic conditions to achieve precise targeting of tumors and their acidic microenvironment.

Nevertheless, immune cell membrane–based nanocarriers universally encounter issues such as donor-to-donor variability, dynamic phenotypic changes, and scalability constraints, which continue to hinder their clinical translation.

Functional integration of immune cell membranes substantially enhances the active targeting capability and transmembrane delivery efficiency of biomimetic liposomes at pathological sites, thereby significantly improving the bioavailability of the encapsulated therapeutics. Moreover, the immune-active components inherently present in the membrane architecture contribute to the modulation of the pathological microenvironment, synergistically augmenting the clinical benefit.

#### Extracellular vesicle resources

Extracellular vesicles (EVs) are membrane-enclosed vesicles secreted by cells that carry various cellular constituents and serve as crucial mediators of intercellular communication.^110–112^ They are ubiquitously distributed across a range of biological fluids, including blood, lymph, urine, and saliva. While EVs and cellular membranes both possess a phospholipid bilayer structure, distinct differences in their membrane composition underlie the specialized functions and biological roles unique to EVs. For instance, EVs encapsulate biomolecules derived from their parent cells, such as tumor-associated antigens in tumor-derived EVs, lipids including SMs and ceramides, as well as various RNA species like miRNAs and mRNAs, all of which are integral to mediating intercellular signaling.^113–115^ According to their biogenetic origin, EVs are commonly categorized into groups such as mammalian cell-derived EVs and bacterial OMVs.^95,96^

Naturally derived EVs possess membrane structures closely resembling those of liposomes, which facilitates their seamless integration *via* membrane fusion to generate multifunctional biomimetic liposomes. Compared to biomimetic liposomes incorporating only cell membranes, those integrating EV components better retain and highlight the distinct biological features of their parent cells. Through the delivery of specific RNAs or proteins, EVs can engage directly with target cells to mediate signal transduction or modulate cellular functions, thereby synergistically augmenting the therapeutic efficacy of encapsulated drugs. For instance, Zhu and colleagues engineered a hybrid nanoplatform (Lip-CExo@PTX) by fusing chimeric antigen receptor (CAR)-T cell-derived EVs with liposomes.^116^ This system leverages CAR-mediated targeting to selectively deliver paclitaxel and cytotoxic granules to tumor cells, enhancing antitumor efficacy and offering a novel approach for immunochemotherapy in lung cancer.

OMVs are naturally secreted structures by bacteria, typically abundant in bacterial surface antigens and receptors such as lipopolysaccharides (LPSs) and glycopeptides, which can activate innate immune responses, particularly through the TLR signaling pathway.^117–119^ Hybrid biomimetic liposomes formed by fusing exogenous OMVs with endogenous liposomes exhibit strong immune activation properties, making them highly effective for vaccine delivery, especially in the development of vaccines against bacterial or viral infections.

## Preparation methods

**Fig. 8:**
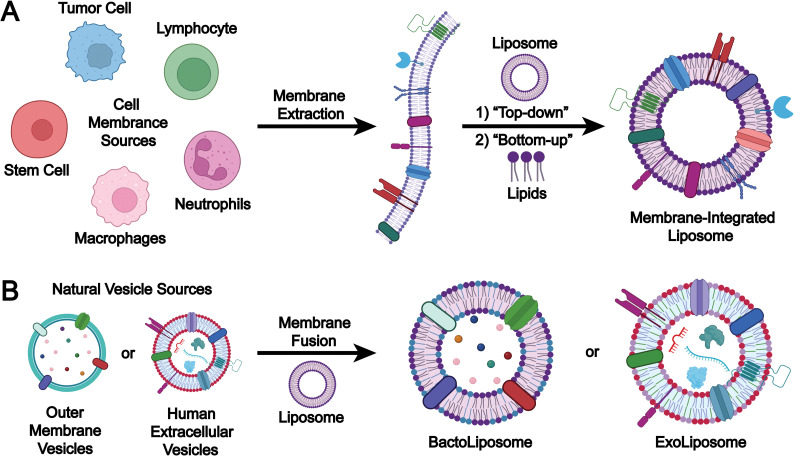
Preparation strategies for biomimetic liposomes: (A) extract and select the cell membrane from purified cells, then incorporate the membrane components onto the surface of liposome membranes using either (1) a top-down or (2) bottom-up strategy to prepare biomimetic liposomes; (B) select naturally derived extracellular vesicles (outer membrane vesicles or mammalian EVs) and use a membrane fusion strategy to prepare various hybrid biomimetic liposomes (*e.g.*, bactoliposomes or exoliposomes). Image created with BioRender.com.

The preparation methods for biomimetic liposomes can be classified according to the origin of membrane components into two main approaches: (1) the cell membrane incorporation method, in which extracted cell membrane components are integrated with liposomes to form structurally unified biomimetic liposomes (Fig. 8A); and (2) the EV membrane fusion method, where membrane components derived from mammalian EVs or microbial OMVs are fused with liposomes to produce hybrid biomimetic liposomes (Fig. 8B).

### Membrane incorporation

The preparation of biomimetic vesicles *via* cell membrane incorporation can be typically summarized into two key steps: (1) cell membrane extraction and (2) cell membrane incorporation.

#### Cell membrane extraction

Cell membrane extraction can be performed using physical methods, chemical methods, or a combination of both.^107,120–123^ Physical methods entail the application of mechanical forces (*e.g.*, ultrasonic vibration or shear stress), temperature fluctuations (*e.g.*, freeze–thaw cycles), or osmotic pressure changes to disrupt the cell membrane and release intracellular components.^122–126^ These methods are favored for their operational simplicity and minimal reliance on exogenous chemical reagents. However, they typically suffer from lower extraction efficiency and carry a greater risk of inducing irreversible damage to membrane structures, particularly membrane-associated proteins. Such damage is primarily attributed to the non-uniform distribution of physical energy, which can lead to structural disruption and consequent loss of protein functionality.

Chemical methods for cell membrane extraction primarily entail detergent solubilization and enzymatic digestion. Compared to physical methods, chemical extraction typically offers higher efficiency, enhanced selectivity, and improved preservation of the native structure and functional integrity of membrane proteins.^127–130^ However, these methods are often more costly, procedurally complex, and may introduce residual reagents that interfere with subsequent purification or downstream applications.

Currently, the integration of physical and chemical approaches enables synergistic advantages, enhancing both the efficiency and selectivity of cell membrane extraction. A typical procedure involves initial mechanical disruption of cells using a Dounce homogenizer, followed by differential centrifugation to remove intracellular organelles, and subsequent chemical treatment with a specialized membrane protein lysis buffer.^131–133^ This combined strategy ensures effective cell lysis while preserving the native conformation and biological activity of membrane-associated proteins.

#### Cell membrane incorporation

Cell membrane incorporation strategies are generally classified into two categories: top-down and bottom-up approaches. The core principle of top-down strategies entails the use of native cell membranes or membrane fragments, which are either assembled with or integrated onto preformed synthetic liposomes *via* methods such as extrusion or sonication to generate biomimetic liposomes. These approaches were originally developed to bridge the gap between synthetic nanocarriers and biological systems. By preserving functional components of the source membrane, such as membrane proteins, glycoproteins, and surface receptors, top-down strategies significantly enhance the biocompatibility, immune evasion, and targeting capacity of liposomal formulations. For instance, red blood cell (RBC) membrane fragments can be incubated with preassembled liposomes at 37 °C for 1 h under gentle agitation to promote membrane embedding, resulting in the formation of RBC-mimetic liposomes, which inherit the immune-evasive properties of native RBCs and can avoid macrophage-mediated phagocytosis.^103,134^

**Fig. 9:**
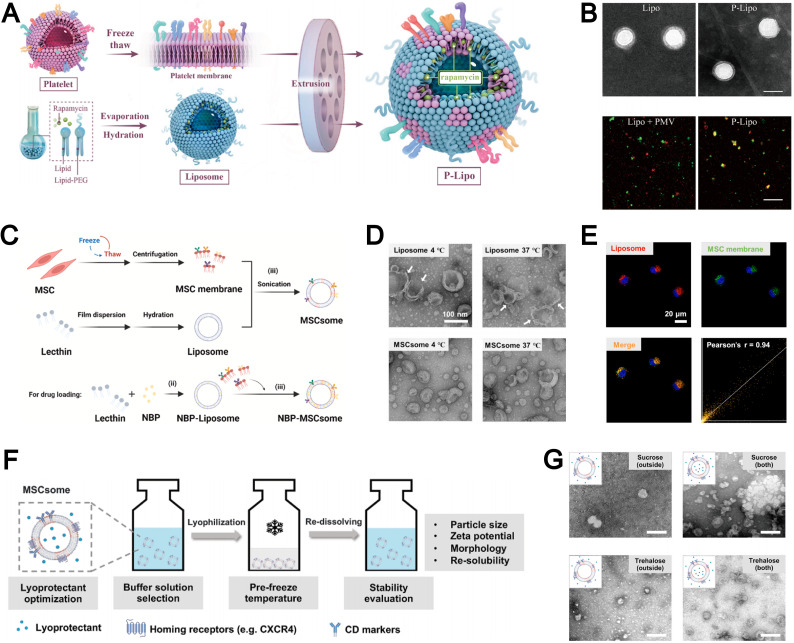
(A) Schematic diagram of the synthetic process of platelet membranes-hybrid biomimetic liposomes P-Lipo. (B) Top: Transmission electron micrographs (TEMs) of liposomes and P-Lipo. Down: Confocal laser scanning microscope (CLSM) images of either a mixture of liposome and platelet membrane vesicle (PMV) or P-Lipo (red: liposome, green: PMV). Reproduced from ref. 100 with permission from Elsevier, copyright (2020). (C) Schematic diagram of the three-step preparation of liposome and mesenchymal stem cell-biomimetic liposomes (MSCsome). (D) Representative TEM images of Liposome and MSCsome after storing at 4 °C and 37 °C for 7 days, respectively. (E) CLSM images of DiO and DiD-double labeled MSCsome in different channels with Pearson's *r* analysis of the colocalization of DiD and DiO. Red signal, DiD-labeled Liposome (DiD-Liposome); green signal, DiO-labeled MSC membrane; blue signal, 4′,6-diamidino-2-phenylindole (DAPI)-labeled nuclei. Reproduced from ref. 135 with permission from Elsevier, copyright (2024). F) Illustration to demonstrate the optimization of the lyophilization of MSCsomes. (G) TEM images of MSCsomes pre-frozen at −20 °C and protected with sucrose and trehalose in the out-layer only, as well as MSCsomes protected with sucrose and trehalose in both the out-layer and the inner-layer. Reproduced from ref. 136 with permission from Wiley-VCH, copyright (2024).

Compared to other nanoparticles, liposomes-with their distinctive phospholipid composition-directly incorporate native cell membrane components into the liposomal bilayer rather than merely enveloping the nanoparticle core with an external coating. For instance, Song *et al.* employed a freeze–thaw technique to extract platelet membranes as lipid building blocks, subsequently integrating them into lipids *via* extrusion to fabricate stable biomimetic liposomes (Fig. 9A).^100^ Concurrently, during liposome formulation, the authors encapsulated the atheroprotective agent rapamycin as a model drug within this biomimetic delivery system, which effectively inhibited atherosclerosis progression while minimizing systemic drug toxicity. The resultant platelet membrane-coated liposomes exhibited physicochemical properties comparable to conventional liposomes (Fig. 9B). Furthermore, the authors further confirmed the efficient incorporation of platelet membrane proteins into the lipid bilayer using confocal laser scanning microscopy (CLSM).

In addition to the extrusion technique, sonication is another widely employed method within the “top-down” approach for the fabrication of biomimetic liposomes. For example, Dong *et al.* demonstrated that sonication could effectively integrate MSC membranes into liposomes to form MSCsomes, a biomimetic nanoplatform for treating cerebral ischemia–reperfusion injury (Fig. 9C).^135^ The MSCsome formulated demonstrated remarkable stability, maintaining consistent morphology and size even after prolonged storage under both high- and low-temperature conditions (Fig. 9D). The authors also employ CLSM to further confirm that the successful integration of mesenchymal stem cell membrane components within the liposomal structure (Fig. 9E). This sonication-based approach provides a simple and efficient strategy for the integration of MSC membranes into liposomes and the ensurance of uniform assembly, high structural stability, and reliable preservation of membrane components.

The “top-down” approach yields biomimetic liposomes that closely resemble natural membranes from various cell types. However, their clinical applicability is hindered by unresolved issues related to long-term storage stability. To address this limitation, Zhang *et al.* investigated the preservation of MSCsome by pre-freezing the formulation at −20 °C in Tris buffer (pH 7.4) supplemented with 10% trehalose, which effectively maintained liposomal integrity for at least 3 months (Fig. 9F).^136^ Importantly, this preservation strategy safeguarded key membrane proteins on the hybrid liposomes and sustained CXCR4-mediated targeting capacity both *in vitro* and *in vivo*. Consequently, the hybrid liposomes exhibited comparable tumor-targeting efficiency to freshly prepared biomimetic liposomes. The authors further observed that post-preparation addition of trehalose and sucrose failed to adequately protect the vesicular structure, whereas inclusion of these cryoprotectants during the lipid film hydration step resulted in well-preserved vesicle morphology (Fig. 9G). Their work demonstrates that incorporating cryoprotectants during formulation enables long-term preservation of biomimetic liposomes while retaining structural integrity and functional targeting, thereby enhancing the practicality of biomimetic nanoplatforms for clinical translation.

The “Bottom-up” and “Top-down” approaches are fundamentally contrasting strategies for biomimetic liposome fabrication. The “Bottom-up” method utilizes lipid molecules, cholesterol, and cell membrane components at the molecular level to self-assemble, gradually forming biomimetic liposomes. This method enables precise control over the particle size, membrane structure, and drug loading capacity of biomimetic liposomes, thereby producing high-performance drug-loaded liposomes and enhancing their delivery efficiency. The development of bottom-up approaches has facilitated the synthesis of bioinspired delivery systems through surface functionalization with ligands and molecules capable of binding to the receptors of specific target cells.

**Fig. 10:**
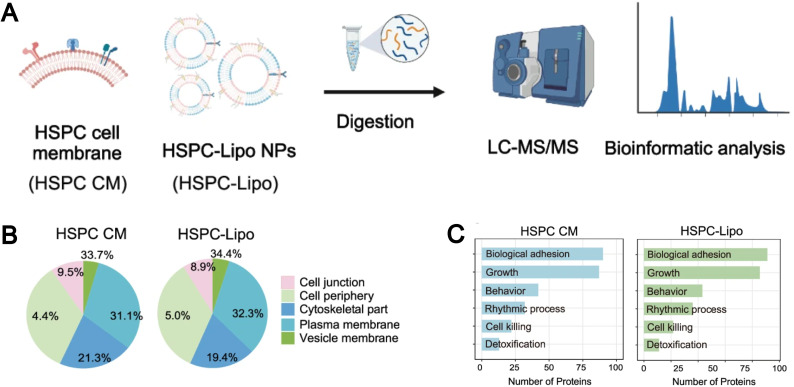
(A) Schematic illustration of liquid chromatography tandem mass spectrometry (LC-MS/MS) sequencing design of HSPC cell membrane and HSPC-Lipo. (B) Protein type analysis diagram. (C) Protein pathway enrichment analysis. Reproduced from ref. 138 with permission from Springer Nature, copyright (2024).

Currently, the thin-film hydration method and the microfluidic method are two widely utilized “Bottom-up” approaches for liposome preparation. The incorporation of cell membrane components during the preparation process enables the fabrication of biomimetic liposomes. For example, Molinaro *et al.* integrated proteins from the leukocyte plasma membrane into the liposomes in the process of preparing liposomes, successfully forming protein–liposomes (leukosomes).^137^ These leukosomes retained the typical multifunctionality and physicochemical properties of the liposome formulation, allowing for preferential targeting of inflammatory blood vessels, selective and efficient delivery of dexamethasone to inflammatory tissues, and reduction of the inflammatory response in local inflammation models. The authors can precisely control the protein-to-lipid ratio to prepare different leukosomes. The author also found that the decrease in the liposome diameter correlated with the increase in protein content in the lipid bilayer, with the protein-to-lipid ratio increasing from 1 : 100 and 1 : 600 to 1 : 300. Proteomic analysis of the leukosome demonstrated that its membrane primarily consists of integral or lipid-anchored proteins, cytoskeletal and junctional proteins, peripheral proteins, as well as vesicular or secreted proteins. Functionally, these proteins participate in a variety of biological processes, including transport, signal transduction, immune response, cell adhesion, lipid metabolism, and structural maintenance. Compared to the “Top-down” approach, biomimetic liposomes prepared using the “Bottom-up” method better preserve the integrity and functionality of the cell membrane protein. For example, Li *et al.* found that biomimetic hematopoietic stem and progenitor cell membrane incorporated liposomes (HSPC-Lipo) prepared using the thin-film hydration method exhibited a similar classification and proportion of protein subtypes as their parent HSPC cell membrane through mass spectrometry analysis (Fig. 10A).^138^ Interestingly, pathway enrichment analysis revealed that despite undergoing multiple freeze–thaw cycles and fusion with liposomes, HSPC-Lipo vesicles retained and enriched gene expressions and signaling pathways like those of the HSPC cell membrane, particularly pathways related to cell adhesion (Fig. 10B and C).

However, during the bottom-up construction of biomimetic liposomes, it is essential to consider the interactions between cell membrane components and the liposomal bilayer, such as the compositional ratio and the strength of interaction. These factors affect not only the size of the biomimetic liposomes but also their recognition by biological systems and their overall functionality. For example, Rahman *et al.* found that the rigid linker (EAAAK)_8_, which adopts a straight conformation and serves to maintain a fixed distance between the NB and the liposomal membrane, restricts the movement of the NB and may reduce its binding efficiency to HER2. Moreover, the hydrophobic and self-cleavable nature of this linker can lead to NB detachment, thereby compromising targeted delivery. To address these issues, they designed a flexible linker (GGGGS)_8_ and a human single transmembrane domain (STMD) to attach the NB to the liposomal surface.^36^ This flexible linker allows greater conformational freedom for the NB, reduces steric hindrance, and thus improves its biological activity. In addition, the resulting interaction can lead to the formation of a protein-based protective layer on the liposome surface, which contributes to improved stability and functionality.

While the “bottom-up” strategy enables fine control over the architecture and function of biomimetic liposomes, its complexity and high cost hinder industrial scalability. Thus, innovative and simplified fabrication methods are urgently needed to support large-scale production.

### Membrane fusion

The membrane fusion method is used to prepare biomimetic liposomes by combining naturally derived EVs with engineered liposomes. This approach enhances the stability, targeting efficiency, and drug-loading capacity of the delivery system. The preparation process typically involves two main steps: (1) EVs are first isolated and purified from cell culture supernatants or biological fluids; and (2) membrane fusion techniques are then applied to merge the EVs with pre-formed liposomes, leading to the formation of biomimetic liposomes.

#### Isolation and purification of EVs

Ultracentrifugation is the gold standard for the isolation and purification of EVs. This technique employs a series of differential centrifugation steps to sequentially eliminate cell debris, large vesicles, and protein contaminants.^139–142^ EVs are ultimately isolated using ultrahigh-speed centrifugation (100 000×*g*), which enables high purity and yield. Despite being time-consuming and potentially causing partial structural damage to EVs, ultracentrifugation remains the mainstream method in EV research. Alternative techniques such as size exclusion chromatography (SEC) and density gradient centrifugation are also frequently employed.^143–145^ Compared with ultracentrifugation, these methods better preserve the biological integrity of EVs and offer improved purity. However, they are often associated with lower recovery rates and face limitations in scalability for industrial applications.

The extraction of natural EVs is complex and labor-intensive, as it relies on ultracentrifugation, size-exclusion chromatography, and other purification techniques. In addition, the high variability in size, membrane protein composition, and cargo further limits their consistency and practical use. To overcome these limitations, exosome mimetics (EMs) have been developed, along with various preparation methods such as mechanical extrusion.^146,147^ EMs can be produced on a large scale with controlled composition, and they retain essential membrane proteins from their parent cells. Furthermore, they possess strong drug-loading capacity and achieve high drug delivery efficiency. As a result, EM-based biomimetic liposomes exhibit broad potential for biomedical applications. Cell extrusion is one of the most widely used methods for EM preparation. In this approach, a large number of EMs are generated by sequentially passing a cell suspension through polycarbonate membrane filters with defined pore sizes.

#### Membrane fusion of EVs with liposomes

Due to inherent membrane–membrane interactions, EVs and liposomes are capable of spontaneous membrane fusion without the need for excessive physical or chemical interventions. However, the spontaneous fusion strategy typically suffers from low efficiency and poor uniformity, limiting its suitability for large-scale applications.

Several physical methods have been adopted to enhance membrane fusion, including freeze–thaw cycles, ultrasonication, and extrusion. For instance, repeated transitions between −80 °C and 37 °C disrupt the membranes of EVs and liposomes, followed by reassembly that facilitates fusion.^148^ Nevertheless, the lack of precise control over the intensity and direction of physical forces frequently leads to damage of membrane structures, which can result in functional loss and drug leakage.

To improve membrane interactions, chemical modification strategies have also been introduced. These methods typically involve the attachment of specific chemical or biological molecules to the surface of EVs or liposomes. For instance, PEG removes the hydration layer from vesicle surfaces, which increases the likelihood of membrane fusion.^149^ Similarly, the modification of EVs with lipophilic molecules such as DSPE-PEG or cholesterol–PEG enhances membrane compatibility. Despite their advantages in fusion efficiency and controllability, chemical approaches have limitations. PEGylation, for example, may alter surface properties, reduce binding to target cells, and impair biological function.

**Fig. 11:**
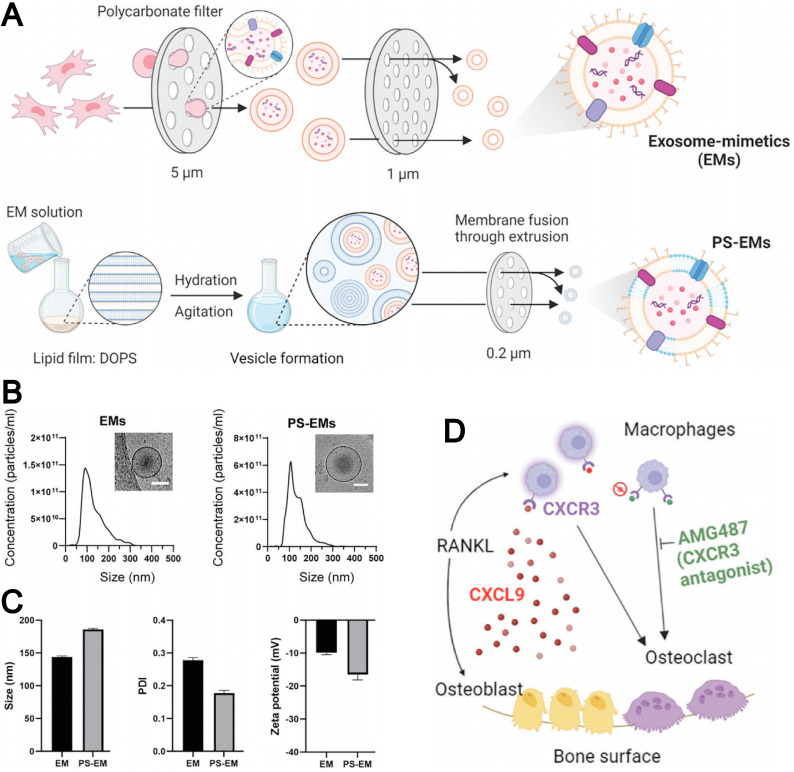
(A) Schematic illustration of the procedure for generating exosome mimetics (EMs) and phospholipid-incorporated EMs (PS-EMs). (B) Size distribution of EMs and PS-EMs revealed by nanoparticle tracking analysis (NTA) with representative cryo-TEM images on top. (C) Size, polydispersity index, and zeta potential of EMs and PS-EMs. (D) Schematic depicting chemokine control of osteoclast recruitment *via* the CXCL9-CXCR3 axis. Receptor activator for NF-kB ligand (RANKL), either directly or indirectly, induces the release of CXCL9 from osteoblast progenitors, which activates macrophages expressing the CXCR3 receptor. This activation triggers macrophage recruitment and differentiation into osteoclasts, contributing to bone resorption. Reproduced from ref. 73 with permission from Wiley-VCH, copyright (2024).

The strategy of preparing biomimetic liposomes through the fusion of EMs with liposomes has attracted increasing attention. For instance, Minjee *et al.* developed EMs derived from mesenchymal stem cells using a cell extrusion technique and introduced PS onto the EM membrane during the fusion process with liposomes (Fig. 11A).^73^ This modification enhanced the binding affinity of the EMs to osteoclast precursors. As shown in Fig. 11B and C, the resulting PS-EMs exhibited an increased particle size compared to unmodified EMs and a reduced zeta potential. PS-EMs were capable of encapsulating AMG487, a CXCR3 receptor antagonist, which was employed to inhibit the migration and activation of osteoclast precursors toward the bone matrix (Fig. 11D). This approach provides a promising strategy for targeting bone-related diseases by enhancing cellular interactions and functional performance in exosome-based delivery systems.

Consequently, the development of improved membrane fusion techniques has become a key research focus, particularly approaches that address the drawbacks of current physical and chemical methods. An ideal fusion strategy should significantly increase both efficiency and uniformity while preserving the structural and functional integrity of EVs. Microfluidic platforms provide a promising solution by enabling precise control over fluid flow and particle behaviours. Adjustments to fluid dynamics or electric fields under such conditions allow for better fusion outcomes without compromising membrane stability.

## Applications

Biomimetic liposomes are primarily applied in drug delivery systems, especially for targeted therapies. In addition, they have shown potential in a variety of other areas, including immune camouflage, imaging, immunomodulation, gene delivery, and vaccine development.

### Targeted therapy

**Fig. 12:**
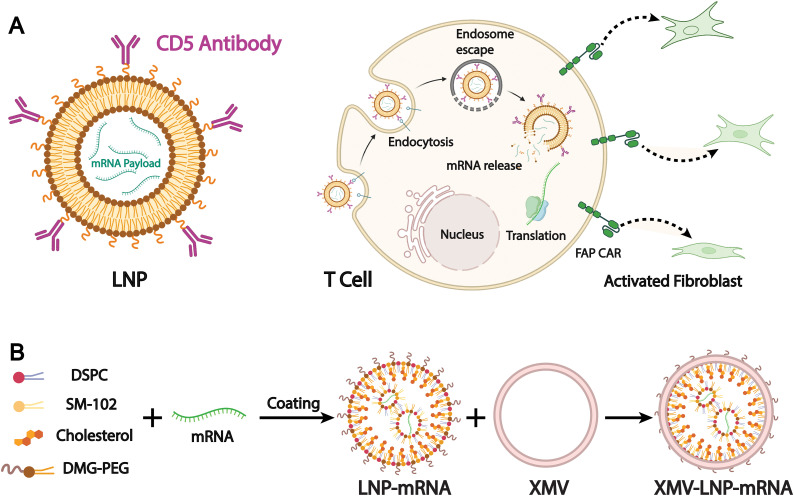
(A) Schematic outlining the molecular process to create transient FAPCAR T cells using CD5-targeted LNPs. Adapted from ref. 152 and created with BioRender.com. (B) A schematic illustration of the preparation of XMV coating on the LNP-mRNA surface to generate XMV-LNP-mRNA. Adapted from ref. 153 2025 and created with BioRender.com.

Targeted therapy is one of the primary objectives in the development of drug delivery systems. Most liposomal drug delivery systems mainly depend on the passive EPR effect to accumulate in pathological tissues like tumors. However, the efficiency of passive targeting remains limited in clinical settings due to physiological barriers, tumor heterogeneity, and inconsistent vascular permeability.^150,151^ To address these limitations, biomimetic liposomes have emerged as a promising alternative. By incorporating components derived from natural cell membranes, such as those from immune cells, cancer cells, or stem cells, biomimetic liposomes are designed to improve biological recognition and tissue-specific accumulation. They are intended to achieve accurate cell targeting in complex physiological systems and to initiate targeted therapeutic intervention or cellular reprogramming. For example, Rurik *et al.* developed a method to deliver modified mRNA *in vivo via* CD5-targeted LNPs, which allows direct generation of antifibrotic CAR T cells inside the body (Fig. 12A).^152^ They successfully induced transient expression of CAR T cells in a mouse model of heart failure, leading to a significant reduction of cardiac fibrosis and restoration of heart function. This work also demonstrates the innovative potential of LNPs in precise and dynamic cell therapy applications.

Although antibody targeting offers high specificity and efficacy, its large size, strong immunogenicity, limited stability, and high cost restrict its use in LNPs. Biomimetic design enables LNPs to partially replace the targeting functions of conventional antibodies. For example, Wang *et al.* designed a novel vaccine delivery system based on xenogeneic cell membrane vesicles (XMVs). They used xenogeneic cell membranes that expose tissue-specific antibodies to mimic the rapid antigen processing and presentation seen in xenotransplant rejection. This system effectively delivers peptide antigens and mRNA-encoded antigens to DCs *via* hybrid XMV-LNPs (Fig. 12B).^153^ The authors compared the therapeutic efficacy of this biomimetic liposomal nanovaccine with the gold-standard SM-102 nanovaccine and found that treatment with XMV-LNP-OVA significantly suppressed tumor growth compared to LNP-OVA treatment. Moreover, immunohistochemistry analysis showed that XMV-LNP-OVA treatment markedly enhanced CD8^+^ T cell infiltration in the tumor tissue. These results indicate that biomimetic XMV-LNP-mRNA can enhance the DC-targeted delivery of LNPs-based mRNA vaccines, significantly improving the antitumor immune responses of the SM-102 vaccine formulation.

**Fig. 13:**
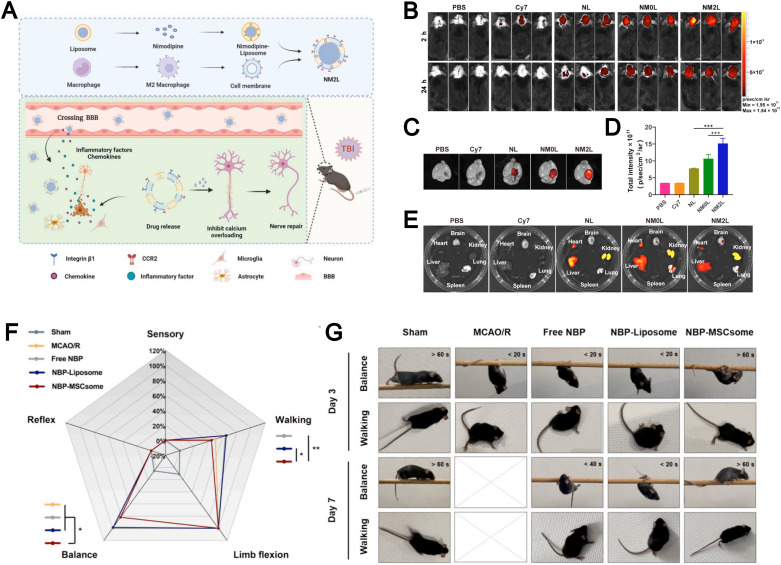
(A) Preparation procedures of NM2Ls and the mechanism for the treatment of traumatic brain injury (TBI). *In vivo* fluorescence imaging of mice. Whole body imaging (B) at 2 h and 24 h post TBI. Brain imaging (C) and fluorescence intensity (D) at 24 h post TBI. Organ imaging (E) at 24 h post TBI (*n* = 3). Reproduced from ref. 104 with permission from Elsevier, copyright (2025). (F) Radar plot reflecting the scores of mice in each component of mNSS evaluation on Day 3, including sensory, reflex, balance, limb flexion, and walking. The higher percentage of the score represents the more severe damage on the behavioral evaluation item of the mice. (G) Behavioral images showing the performance of mice regarding balance and walking on Day 3 and Day 7. Reproduced from ref. 135 with permission from Elsevier, copyright (2024).

Liposomes face significant challenges in treating brain-related diseases such as traumatic brain injury (TBI) and ischemic stroke because the blood–brain barrier (BBB) limits their brain penetration and non-specific accumulation in other organs lowers targeting efficiency. The emergence of biomimetic liposomes has helped overcome some of these difficulties. For example, Zhang *et al.* designed M2 macrophage membrane-hybrid biomimetic liposomes loaded with nimodipine (NM2Ls), a Ca^2+^ influx inhibitor (Fig. 13A).^104^ Experimental results demonstrated that intravenous administration of NM2Ls allowed them to evade immune clearance and to target the brain through CCR2 (Fig. 13B–D), while reducing liposome accumulation in other organs (Fig. 13E), which significantly alleviated brain inflammation in a TBI mouse model. The authors confirmed that NM2Ls prepared by employing a biomembrane strategy could act as promising brain-targeted therapeutics with considerable potential. Their study highlights that leveraging M2 macrophage membranes to construct biomimetic liposomes offers an effective strategy to achieve brain-targeted delivery and underscores the therapeutic potential of biomimicry in treating neurological diseases.

Similarly, Dong *et al.* engineered biomimetic liposomes by integrating mesenchymal stem cell (MSC) membranes with liposomes for the treatment of ischemic stroke.^135^ These MSCsomes efficiently delivered dl-3-*n*-butylphthalide (NBP) to the injured hemisphere. Notably, mice receiving NBP-MSCsomes exhibited a marked improvement in modified neurological severity score (mNSS) by day 3, showing better balance and walking ability, which indicated neuronal recovery during the acute inflammation phase of ischemia–reperfusion injury (Fig. 13F). Nearly half of these mice maintained their balance on the beam for over 60 seconds and showed a tendency to walk normally without signs of paralysis (Fig. 13G). The incorporation of MSC membranes in biomimetic liposomes enables precise brain-targeted delivery, enhancing therapeutic efficacy while reducing off-target accumulation.

Biomimetic liposomes outperform traditional antibody-modified systems in targeted therapy. Despite ongoing challenges in safety and manufacturing, they possess significant potential as a foundational technology for precision medicine.

### Immune camouflage

Immune camouflage is also a notable application of biomimetic liposomes. Liposomes incorporated with immune cell membranes, such as those from neutrophils, macrophages, or NK cells, help them evade immune detection. As a result, these liposomes can deliver drugs more effectively to tumors or diseased tissues while minimizing toxicity to healthy cells. For example, Pitchaimani *et al.* developed a fusogenic liposomal system called “NKsome,” which incorporates NK cell membranes.^97^ These membranes naturally participate in immune surveillance and facilitate tumor targeting. The resulting NKsome retained key membrane proteins responsible for cellular recognition and demonstrated strong affinity toward breast cancer cells.

**Fig. 14:**
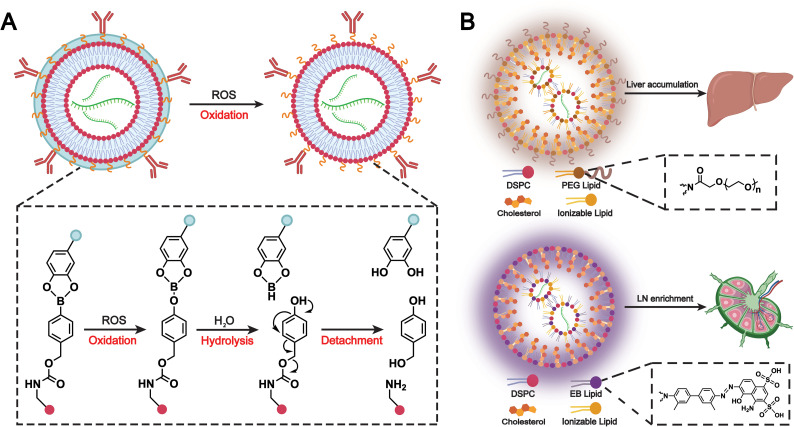
(A) Schematic illustration of the ROS responsiveness of Plofsomes and the detachment of 4-arm PEG-oDPs. Plofsomes turn into a fusogenic state from a non-fusogenic state. Adapted from ref. 154 and created with BioRender.com. (B) Comparative analysis of composition and biological function. Due to the incorporation of PEG-lipid, traditional PEG-LNP results in accumulation in liver tissues after i.m. injection. By contrast, the introduction of EB-lipid in the EB-LNP system alters the biodistribution pattern of the mRNA vaccine, leading to enrichment in LNs. Adapted from ref. 156 and created with BioRender.com.

In the previous section, we discussed how chemokine-mediated targeting can enhance the accumulation of biomimetic liposomes at sites of inflammation or tumors. However, some normal tissues and other inflamed areas also express chemokine receptors, which may cause liposome accumulation in non-target tissues and compromise safety. To address this issue, Zhao *et al.* developed a polymer-locking fusogenic liposome (Plofsome), which not only crosses the BBB but also incorporates a “lock” mechanism that enables selective fusion.^154^ The “lock” utilizes a traceless reactive oxygen species (ROS)-cleavable linker, ensuring that fusion occurs only after reaching glioblastoma tissues with elevated ROS levels (Fig. 14). Ultimately, the system delivers short interfering RNA or CRISPR–Cas9 ribonucleoprotein complexes into the cytoplasm of glioblastoma cells. This immune camouflage strategy effectively prevents the accumulation of liposomes in non-target cells, which enhances the safety of combined RNAi and CRISPR–Cas9 therapies and leads to a significant prolongation of survival in LN229R glioma-bearing mice.

Conventional PEGylated liposomes provide immune camouflage to prolong systemic circulation.^85^ However, their hydrophobicity and structural stability often result in hepatic recognition and accumulation, limiting the efficacy and safety of mRNA vaccines.^155^ To address this issue, Feng *et al.* have developed a biomimetic Evans blue-modified lipid nanoparticle (EB-LNP) system with high affinity for albumin, the most abundant protein in human lymphatic fluid (Fig. 14B).^156^ Since albumin is transported unidirectionally from intramuscular blood capillaries into interstitial tissues and primarily recollected by the lymphatic system, this albumin-recruiting strategy facilitates efficient lymphatic drainage and changes the systemic distribution of nanoparticles. By the formation of an endogenous albumin corona, EB-LNPs achieve a biomimetic self-camouflage effect that enables preferential transport to draining lymph nodes while avoiding hepatic accumulation. This approach significantly enhances dendritic cell uptake and antigen presentation, elicits robust cellular and humoral immune responses, and provides a promising platform for the safe and effective delivery of mRNA vaccines.

### Imaging

Bioimaging has become an essential tool for early disease diagnosis, therapeutic monitoring, and real-time evaluation of treatment responses.^157–160^ Despite significant progress, conventional imaging probes are often hindered by poor *in vivo* stability, limited targeting specificity, and rapid clearance by the immune system, thereby restricting their clinical translation.

**Fig. 15:**
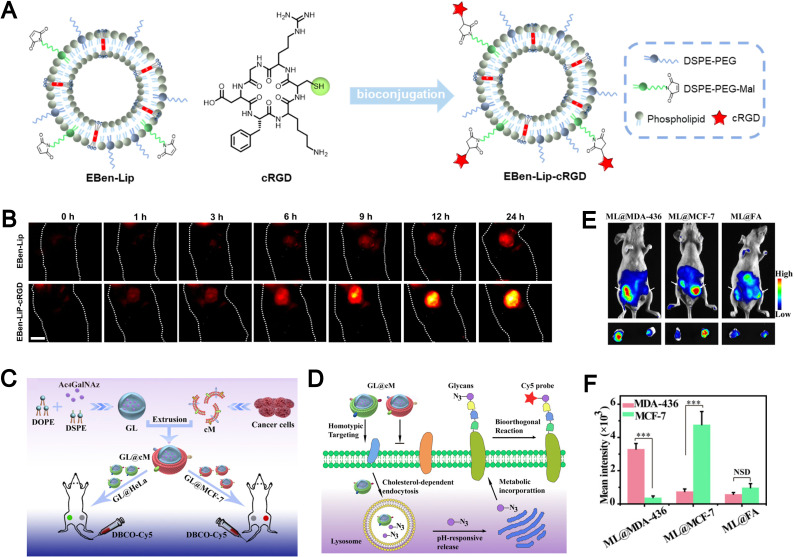
(A) Schematic representation detailing the synthesis of the active targeting fluorescent liposome, EBen-Lip-cRGD, derived from the fluorescent liposome EBenLip. (B) *In vivo* NIR-II imaging of subcutaneous tumor-bearing mice at various time intervals post intravenous administration of EBen-Lip and EBen-LipcRGD. Reproduced from ref. 88 with permission from Wiley-VCH, copyright (2024). (C) Illustration of the construction of biomimetic Ac4GalNAz liposomes (GL@cM) for *in vivo* progenitor cell-selective glycan imaging. The left tumor is HeLa and the right is MCF-7. (D) Scheme of the path of GL@cM for homotypic cell-selective metabolic glycan labeling. (E) *In vivo* fluorescence visualization of mice with tumors administered with ML@MDA-43, ML@MCF-7, and ML@FA on 4 consecutive days. DBCO-Cy5 was intravenously injected into mice on day 5. (F) Quantitative analysis of the intensity from whole-body fluorescence imaging. Reproduced from ref. 161 with permission from the National Academy of Sciences, copyright (2021).

Biomimetic liposomes, endowed with favorable disease-targeting and immune-evasive characteristics, have emerged as promising platforms in the field of biomedical imaging. To enhance tumor-specific accumulation, click chemistry has been employed to conjugate the tumor-targeting peptide cRGD onto the surface of fluorescent probe–loaded liposomes, thereby improving their active targeting ability (Fig. 15A).^88^ While passive accumulation *via* the EPR effect contributes to tumor localization, the incorporation of cRGD further enhances targeting precision. *In vivo* imaging studies demonstrated that mice treated with EBen-Lip-cRGD exhibited significantly stronger NIR-II fluorescence at tumor sites compared to non-modified controls (Fig. 15B). These results highlight the potential of fluorescent liposomes modified with tumor-targeting peptides (such as cRGD) as efficient imaging agents for tumor visualization.

The presence of multiple membrane receptors on the surface of biomimetic liposomes confers them with high selectivity toward homologous tumor cells. For example, Liu *et al.* developed pH-responsive azidosugar-loaded liposomes cloaked with natural cancer cell membranes to achieve tumor cell–selective glycan engineering (Fig. 15C).^161^ This membrane camouflage strategy effectively inhibited protein corona formation and reduced macrophage-mediated clearance, thereby enhancing *in vivo* metabolic glycan labeling. The receptor-rich membrane coating also facilitated improved cellular uptake and labeling efficiency (Fig. 15D). *In vivo* imaging studies further demonstrated that biomimetic liposomes, ML@MDA-436 and ML@MCF-7, achieved 5.6-fold and 4.8-fold stronger tumor targeting toward MDA-MB-436 and MCF-7 cells, respectively, compared to conventional folic acid-modified liposomes (ML@FA) (Fig. 15E and F). Biomimetic liposomes enhance tumor-specific accumulation, demonstrating the advantage of biomimicry for precise and targeted imaging.

Biomimetic liposomes have emerged as a versatile platform that integrates both diagnostic and therapeutic functions, thereby advancing the field of theranostics—simultaneous disease detection and treatment. By closely mimicking the surface properties of natural cell membranes, these nanocarriers achieve immune evasion, prolonged circulation times, and active targeting of diseased tissues. Incorporation of various imaging agents, such as fluorescent probes, magnetic resonance contrast agents, or radionuclides, alongside therapeutic payloads, including chemotherapeutics, gene-editing complexes, or immunomodulatory molecules, enables precise spatiotemporal control over drug delivery while facilitating real-time monitoring of therapeutic efficacy. This dual functionality offers distinct advantages in managing complex pathologies, including malignancies, cardiovascular disorders, and neurodegenerative diseases, where early diagnosis and personalized intervention are critical for improved outcomes. The continued development of biomimetic theranostic nanoplatforms thus exemplifies the shift toward precision medicine and underscores their significant translational potential in clinical applications.

### Immunomodulation

Biomimetic liposomes with integrated membrane components have emerged as promising tools for immune regulation and inflammation therapy. Inspired by natural intercellular interactions, these systems replicate the immune evasion and targeting properties of native cells. For instance, Xu *et al.* developed biomimetic liposomes coated with fusion membranes from M2 macrophages and exosomes.^35^ The liposomes achieve drug delivery to inflamed joints through immune surveillance evasion, targeted cell binding, and lysosomal escape. These M2 macrophage-mimetic liposomes provide efficient elimination of urate and peroxides. Moreover, the treatment induces a remodeling of the inflammatory immune microenvironment both inside and outside cells through M1 to M2 macrophage repolarization, cytokine modulation, and ROS clearance. This remodeling supports the reconstruction of the immunological barrier.

In addition, Ma *et al.* developed macrophage membrane-integrated liposomes that served as carriers for carbon dot nanozymes with superoxide dismutase-like activity.^98^ These liposomes successfully delivered the therapeutic agents to inflamed colonic tissue, where they modulated ROS levels and alleviated oxidative stress within the intestinal microenvironment. In a dextran sulfate sodium-induced ulcerative colitis model, treatment with this system led to notable reductions in vascular congestion and mucosal ulceration, along with restoration of vascular architecture. These findings underscore the role of membrane-coated biomimetic liposomes in enhancing the therapeutic performance of nanozymes and broadening their application in inflammatory diseases.

In oncology, integrated membrane-fused liposomes have shown considerable potential for preventing postoperative tumor recurrence. Despite surgical resection being the primary method for treating solid tumors, recurrence remains a major challenge due to residual malignant cells. Ning *et al.* introduced a hybrid liposomal platform constructed from tumor cell membranes, co-loaded with an aggregation-induced emission photosensitizer and metformin.^162^ The tumor-derived membrane allowed for homotypic targeting and selective accumulation at tumor sites. Upon administration, the system initiated photodynamic therapy that caused immunogenic cell death and activated a robust antitumor T cell response. In addition, metformin released from the liposomes promoted the differentiation of T cells into central memory subsets, which contributed to sustained immune protection and reduced the likelihood of tumor relapse.

Stem cell membrane-based biomimetic liposomes have emerged as a promising platform for immunomodulation, particularly regarding inflammation regulation. This capability mainly derives from the intrinsic properties of MSCs, which exhibit anti-inflammatory and immunosuppressive functions. When liposomes receive coatings from MSC-derived membranes, they acquire key membrane proteins such as ICAM-1, CD47, and PD-L1.^48,135,136,139^ These proteins enable immune evasion, reduce macrophage uptake, and modulate immune effector cell activity. In addition, these vesicles interact with specific adhesion molecules and inflammatory receptors, which facilitates their preferential accumulation at inflammation sites.

**Fig. 16:**
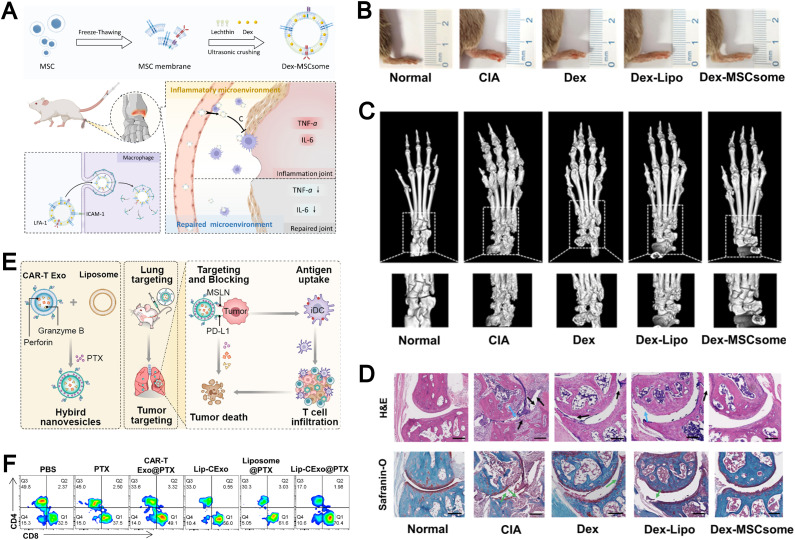
(A) Schematic diagram of rheumatoid arthritis (RA) treatment using mesenchymal stem cell (MSC) membrane-based bionic carriers. (B) Morphology of the right hind paw of the five groups on day 53. (C) Micro-CT images of the right hind paw of the five groups on day 53. (D) Hematoxylin and eosin (H&E) staining and safranin O-fast green staining in the five groups. Black arrow indicates synovial hyperplasia, blue indicates inflammatory cell infiltration, green arrow indicates cartilage destruction, bar: 100 µm. Reproduced from ref. 163 with permission from the American Chemical Society, copyright (2024). (E) Schematic illustration of hybrid nanovesicles of bispecific CAR-T cell-derived exosomes and liposomes for lung cancer chemical-immunotherapy. (F) Representative flow cytometry plots of mature DCs in the tumor microenvironment for different treatment groups (*n* = 3). Reproduced from ref. 116 with permission from the American Chemical Society, copyright (2023).

Based on these underlying mechanisms, researchers developed MSC-derived biomimetic liposomes (MSCsome) through membrane-liposome fusion strategies. For example, Ma *et al.* applied MSCsomes for the targeted delivery of dexamethasone in a murine model of rheumatoid arthritis.^163^ The targeting effect mainly relied on the interaction between lymphocyte function-associated antigen-1 (LFA-1) present on immune cells and intercellular adhesion molecule-1 (ICAM-1) preserved on the MSC membrane (Fig. 16A). This design significantly increased drug accumulation within inflamed joints, which effectively suppressed local inflammation and protected cartilage. Specifically, as shown in Fig. 16B, the Dex-MSCsome treatment group exhibited paw swelling reduction comparable to the healthy control group. Histological analyses revealed that Dex-MSCsomes markedly reduced synovial hyperplasia and inflammatory cell infiltration and preserved cartilage integrity while preventing ankle bone erosion (Fig. 16C and D). In contrast, treatment with free dexamethasone or dexamethasone-loaded conventional liposomes failed to fully alleviate joint lesions. These results highlight the potential of MSC membrane-coated liposomes as a versatile and effective approach for targeted therapy in inflammatory and autoimmune disorders.

In addition to MSC membrane-based biomimetic liposomes that modulate inflammatory microenvironments, CAR-T cell-derived exosome-liposome hybrids further exemplify the integration of immune regulation and targeted chemotherapy.^116^ For example, Zhu *et al.* engineered biomimetic liposomes that integrate tumor-specific CARs and immune checkpoint blockade, along with lung-targeted paclitaxel delivery (Fig. 16E). Notably, the ratio of CD8+/CD4+ T cells in the Lip-CExo@PTX group reached 3.9, significantly higher than in the PTX (0.9), CAR-T Exo@PTX (1.5), Lip-CExo (1.8), and Liposome@PTX (2.0) groups, indicating enhanced infiltration of cytotoxic CD8+ T cells (Fig. 16F). These results demonstrate that Lip-CExo@PTX effectively promotes CD8+ T cell infiltration and activation within the tumor microenvironment, contributing to its superior antitumor efficacy.

Collectively, these examples demonstrate the versatility of integrated biomimetic liposomes in targeting diseased tissues, enhancing immunotherapeutic effects, and enabling long-term disease control. Such systems offer significant promise for advancing precision nanomedicine and addressing challenges under complex pathological conditions.

### Gene delivery

Gene therapy represents a promising strategy for the treatment of a wide range of diseases through the modulation of gene expression at the molecular level. However, its clinical translation remains limited by the difficulty of achieving efficient cellular uptake, safe intracellular trafficking, and effective gene expression of nucleic acid-based therapeutics such as siRNA, mRNA, and CRISPR–Cas9 systems.

In recent years, liposomes, particularly LNPs have gained attention as a class of non-viral delivery vehicles with distinct advantages over traditional viral vectors.^68,69,86,105,153,164–166^ These systems primarily enhance the stability of nucleic acids and improve their targeting efficiency. Such characteristics make them attractive candidates for the efficient delivery of nucleic acids in gene therapy.

**Fig. 17:**
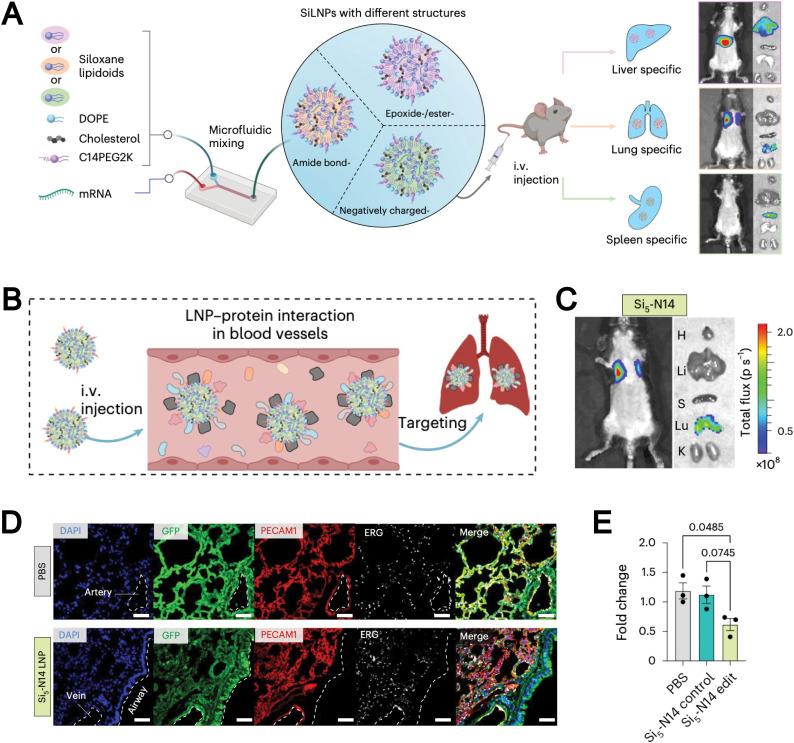
(A) SiLNPs were formulated using a microfluidic mixing device each with a siloxane-incorporated lipidoid, helper lipid (DOPE), cholesterol and PEG-lipid (C14PEG2K). The resulting SiLNPs with different siloxane-incorporated lipidoid structures mediate *in vivo* tissue-specific mRNA delivery to the liver, lungs and spleen. (B) Schematic representation of the interaction of Si5-N14 LNPs with proteins in blood vessels. (C) Luciferase expression imaging from Si5-N14 LNPs 6 h post-injection (FLuc mRNA, 0.3 mg kg^−1^). (D) Representative immunostaining showed GFP knockout in lung ECs. (E) RT-qPCR analysis of GFP in sorted ECs. Reproduced from ref. 70 with permission from Springer Nature, copyright (2025).

For example, Xue *et al.* designed a series of structurally diverse siloxane-based ionizable lipidoids and formulated siloxane-containing lipid nanoparticles (SiLNPs), which not only enhanced the stability of mRNA but also enabled the modulation of its *in vivo* delivery to specific organs such as the liver, lungs, and spleen (Fig. 17A).^70^ Although these SiLNPs are not composed of natural lipids, they can be classified as mechanistically biomimetic liposomes. By regulating behaviors such as endocytosis, organ-specific accumulation, and endothelial penetration, they emulate the homing capabilities of natural nanoparticles or pathogens. For example, the authors found that incorporating siloxanes altered the *in vivo* behavior of lipid nanoparticles, including protein adsorption in blood vessels and interactions with cell membranes (Fig. 17B), which resulted in selective enrichment in tissues such as the liver and lungs (Fig. 17C). To assess gene editing efficacy, the authors co-delivered Cas9 mRNA and GFP sgRNA using Si5-N14 LNPs for CRISPR–Cas9–mediated genome editing in the lungs. Immunostaining revealed a marked reduction in GFP signals in endothelial cells of the pulmonary microvasculature (Fig. 17D). Subsequently, endothelial cells were isolated from lung tissues, and RT–qPCR analysis confirmed a significant decrease in GFP expression following SiLNP-mediated gene editing (Fig. 17E). From a gene delivery perspective, the emulation of natural nanoparticle behaviors by siloxane-containing lipid nanoparticles enables organ-specific targeting and efficient intracellular delivery, illustrating how mechanistic biomimicry can enhance precision and efficacy in therapeutic gene editing.

The development of biomimetic liposomes designed to improve the pharmacokinetics, tissue selectivity, and intracellular availability of nucleic acid therapeutics offers a promising path forward. For example, Nai *et al.* developed a novel therapeutic system by fusing thermosensitive liposomes with macrophage membranes and modifying the surface with tumor-targeting cyclic Arg-Gly-Asp (cRGD) peptides and cell-penetrating peptides, aiming to deliver BCL-2 siRNA specifically to HepG2 liver cancer cells.^105^ By retaining membrane proteins from macrophages, the system reduced uptake by macrophages while enhancing internalization by HepG2 cells. As a result, siRNA preferentially accumulated at the tumor site with minimal distribution to other organs. When combined with hyperthermia, this system achieved significant inhibition of tumor growth.

These systems have shown great potential to enhance the efficiency and safety of gene delivery, thereby facilitating the clinical translation of gene therapies and mRNA-based treatments.

### Vaccine development

The rapid advancement of mRNA-based technologies, particularly in vaccine development, has underscored the critical need for efficient and safe delivery systems. Unlike DNA-based vaccines, mRNA vaccines do not require nuclear entry and eliminate the risk of genomic integration. Nevertheless, their clinical success largely depends on the use of nanocarriers, especially LNPs, to deliver mRNA into the cytoplasm, where it is translated into immunogenic proteins that initiate protective immune responses.

LNP-encapsulated mRNA vaccines have emerged as a powerful platform for the prevention of infectious diseases, as demonstrated by the successful application of SARS-CoV-2 mRNA vaccines. To minimize immune recognition and prevent excessive inflammation, researchers often modify mRNA with nucleosides. However, such modifications significantly reduce innate immune responses, which are essential for the induction of strong adaptive immunity. To address this limitation, Han *et al.* developed a novel LNP component, an adjuvant lipidoid, to improve the immunostimulatory properties of mRNA-LNP vaccines. By replacing part of the ionizable lipids with adjuvant lipidoids, the researchers achieved not only enhanced mRNA delivery but also the acquisition of Toll-like receptor 7/8 (TLR7/8) agonistic activity, which resulted in a stronger innate immune response to the SARS-CoV-2 mRNA-LNP vaccine.^69^ After mice received two vaccine doses, the formulation containing the adjuvant lipidoid (C12-113/TLRa LNP) induced a significantly greater immune response than that induced by the original C12-113 LNP. This response included a higher number of RBD-specific CD8^+^ T cells that expressed Th1-type cytokines such as IFN-γ, IL-2, and TNF-α, as well as cytotoxic markers like CD107α. In comparison with C12-113 LNP, the C12-113/TLRa formulation also resulted in a substantial increase in the proportion of double- and triple-positive CD4^+^ and CD8^+^ T cells, reflecting a more robust and polyfunctional T cell response. The incorporation of adjuvant lipidoids into LNPs allows them to mimic the immunostimulatory features of natural pathogens. This mimicry increases innate and adaptive immune responses and shows how biomimicry can improve the design of more effective mRNA vaccines.

Activation of innate immunity can markedly improve the response efficiency of nanoparticle vaccines. However, excessive immune activation has become a major barrier to their broader clinical application. For instance, overstimulation of T cells may result in T cell exhaustion and cytokine storms. To address this issue, Zhai *et al.* designed a biomimetic cascade-targeting nanosystem, termed siRNA@PLOV, which consists of photothermal-sensitive liposomes (PTSLs) fused with attenuated Salmonella outer membrane vesicles (OMVs) to enable precise targeting of tumor tissues and intratumoral T cells.^96^ This fusion strategy allowed the PLOVs to preserve the biological properties of OMVs while simultaneously improving their capacity for drug encapsulation. The authors demonstrated that both the immunogenic nature of OMVs and the photothermal effect contributed to an increased level of T cell infiltration and reversal of the immunosuppressive tumor microenvironment. At the same time, this approach mitigated the adverse effects associated with excessive T cell activation. Flow cytometry results confirmed that treatment with siRNA@PLOV led to the establishment of long-term immune memory in mice. The biomimetic design of siRNA@PLOV, which integrates OMV properties with photothermal-sensitive liposomes, enables precise tumor and T cell targeting, enhances immune activation in a controlled manner, and establishes long-term immune memory, illustrating the advantage of biomimicry for safe and effective nanoparticle vaccines.

**Fig. 18:**
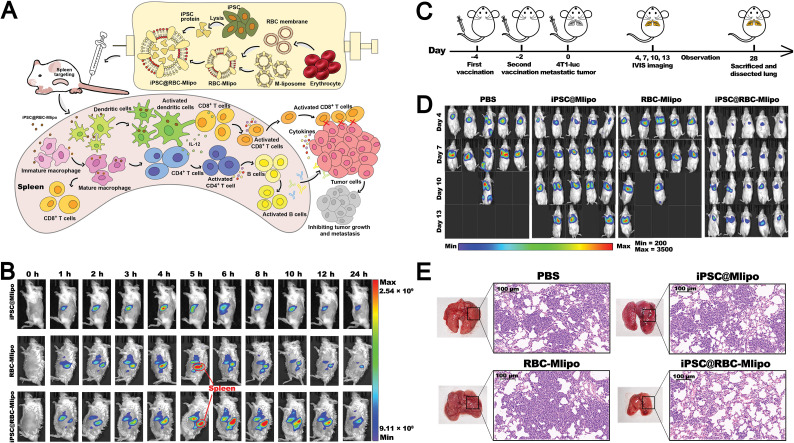
(A) Schematic illustration of the nanovaccine iPSC@RBC-Mlipo design and the immune process *in vivo*. (B) Fluorescence imaging of ICR mice for hours after intravenous injection of group 1 iPSC@Mlipo, group 2 RBC-Mlipo, and group 3 iPSC@RBC-Mlipo. (C) Schematic illustration of setting a 4T1-luc pulmonary metastasis model after vaccinating different groups twice. (D) *In vivo* bioluminescence imaging of 4T1-luc lung metastasis in different groups. The fluorescence signal is expressed in the form of counts. The fluorescence signal is expressed in the form of radiant efficiency. (E) Dissected lungs in groups and images of H&E-stained lung slides after vaccinating different groups. Reproduced from ref. 167 with permission from the American Association for the Advancement of Science, copyright (2021).

Currently, tumor vaccines still face challenges such as the lack of versatility and effective immune induction. To overcome these issues, particularly the problem of insufficient drug loading capacity, Zhai *et al.* developed biomimetic liposomes fused with erythrocyte membranes using modified lipid materials. This nanovaccine encapsulates induced pluripotent stem cell (iPSC) proteins, targets the spleen, and robustly activates systemic tumor-specific immunity (Fig. 18A).^167^ The authors found that RBC-Mlipo and the nanovaccine iPSC@RBC-Mlipo, after fusion with erythrocyte membranes, showed obvious accumulation in the spleen 2 hours after intravenous injection, and the fluorescence signal could be continuously monitored for up to 10 hours (Fig. 18B). To evaluate the antimetastatic potential of the nanovaccine iPSC@RBC-Mlipo, the authors intravenously injected 4T1-luc cells into mice after two vaccinations with iPSC@RBC-Mlipo to mimic the metastatic process of cancer cells (Fig. 18C). *In vivo* bioluminescence imaging showed that, compared with mice in the PBS group, iPSC@Mlipo group, and RBC-Mlipo group, those vaccinated with iPSC@RBC-Mlipo exhibited weaker fluorescence signals, indicating that the nanovaccine could further delay tumor growth and metastasis (Fig. 18D). The authors also found that the antimetastatic effect of the nanovaccine was reflected in fewer pulmonary tumor nodules and more normal lung volumes observed after the mice were sacrificed. H&E staining further confirmed these findings by revealing structural differences in pulmonary nodules among the groups (Fig. 18E). The biomimetic fusion of erythrocyte membranes with liposomes allows targeted delivery of iPSC proteins to the spleen, activates systemic tumor-specific immunity, and inhibits tumor growth and metastasis, highlighting the advantage of biomimicry for the design of potent and organ-targeted nanovaccines.

## Summary and outlook

In this review, we summarize recent progress in the field of biomimetic liposomes, with a particular emphasis on their design principles, preparation techniques, and current applications as an emerging drug delivery platform. Compared with conventional liposomes, biomimetic liposome-based delivery systems possess lower immunogenicity, improved targeting efficiency, and multifunctional characteristics. These advantages have stimulated growing interest in their biomedical applications. To enhance the pharmacokinetics, biodistribution, and therapeutic efficacy of encapsulated agents, researchers have employed strategies such as phospholipid optimization, surface modification, and incorporation of cell membrane components, all intended to increase their utility in clinical settings.

### Challenges and potential solutions

Despite notable advances in recent years, several key challenges continue to hinder the clinical translation of biomimetic liposomes:

#### Stability

The incorporation of functional molecules or cell membrane components may compromise the physical stability of biomimetic liposomes. This disruption can increase the risk of phase separation or aggregation under physiological conditions, which may result in off-target drug release and unintended toxicity to healthy tissues. As such, a comprehensive understanding of how these components interact with the lipid bilayer is essential prior to formulation.

To optimize the stability of biomimetic liposomes, it is necessary to precisely control membrane protein or ligand concentration, type, and spatial distribution to ensure that they are arranged uniformly within the lipid bilayer.^137,138^ This strategy ensures the preservation of the membrane's biological properties while providing structural integrity and fluidity, which results in improved biomimetic behavior of the liposomes and a reduced risk of aggregation or non-specific drug release.

In addition, characterization and theoretical calculations, including molecular dynamics simulations, differential scanning calorimetry (DSC), dynamic light scattering (DLS), and transmission electron microscopy (TEM), provide valuable insights into bilayer stability, membrane protein structure, and lipid–functional molecule interactions, which serve to direct formulation optimization and improve stability and overall performance.

#### Long-term storage instability

Biomimetic liposomes that incorporate natural membrane components are inherently prone to physical and chemical instability. Under ambient or refrigerated conditions, they may degrade, aggregate, or fuse, which disrupts the lipid bilayer and compromises membrane-associated proteins. These instabilities can lead to premature or non-specific drug release, impair targetability, and reduce therapeutic efficacy. In addition, bioactive molecules embedded within the membrane, such as proteins and ligands that mediate cell-specific recognition, remain vulnerable to denaturation or loss of activity over time.

The stability of biomimetic liposomes during storage can be enhanced through tailored strategies depending on the preservation conditions. For short-term storage at 4 °C, optimization of lipid composition with high phase-transition-temperature lipids or cholesterol, adjustment of buffer pH and ionic strength, and the application of surface modifications such as PEGylation can maintain membrane integrity and reduce aggregation.^168,169^ For long-term preservation, lyophilization combined with appropriate protective excipients and optimized freeze-drying conditions, including controlled freezing and residual moisture management, enables the maintenance of lipid bilayer architecture and embedded biomolecules, and facilitates reliable storage and subsequent reconstitution.^136^

Collectively, these strategies provide a framework to improve the storage stability of biomimetic liposomes across various temporal scales, and they support their practical application and clinical translation.

#### Batch consistency and reproducibility

The use of natural cell membranes, such as those from cancer, immune, or stem cells, can lead to batch-to-batch variability in membrane composition and protein content, which affects liposome size, surface characteristics, and cellular interactions. Such variability not only complicates quality control and reproducibility but also introduces uncertainty in pharmacokinetics, biodistribution, and therapeutic outcomes, and it results in significant challenges for large-scale production and clinical translation.

Future efforts to address batch consistency in biomimetic liposomes may focus on standardized cell sources, scalable membrane isolation protocols, and hybrid formulations that combine natural and synthetic components. The integration of automated production platforms with rigorous quality control will be critical to ensure reproducibility and to accelerate clinical translation.

#### Drug loading

Regardless of whether the system is derived through a “top-down” or “bottom-up” approach, the introduction of cell membrane elements often leads to drug leakage. This leakage not only compromises the encapsulation efficiency but also causes premature release, which reduces therapeutic efficacy and increases the risk of off-target effects. Consequently, biomimetic liposomes tend to exhibit reduced drug-loading capacities compared with traditional liposomes, especially in the case of hydrophobic drugs that are less efficiently encapsulated. These limitations remain a major obstacle to clinical translation, as insufficient drug encapsulation restricts both the adjustment of dose and the achievement of therapeutic outcomes.

The combination of both preparation approaches, or the development of alternative methods such as covalent conjugation of hydrophobic drugs to phospholipids, may enhance loading efficiency.^20^

#### Immune response

Biomimetic liposome-based nanovaccines that include antigens or PAMPs have the potential to provoke abnormal immune responses, which may cause irreversible harm. Therefore, thorough safety and toxicity evaluations are required before proceeding with the clinical application of such systems.

To address these challenges, strategies may involve the optimization of antigen/PAMP dosage and release profiles, the incorporation of immunomodulatory agents and the selection of low-immunogenicity membrane sources, comprehensive safety evaluation in both *in vitro* and *in vivo* models, and the design of controllable or environment-responsive systems for precise immune regulation.

#### Unclear release mechanisms

Most current biomimetic liposomes rely on passive drug release, which limits their ability to achieve precise and controlled delivery *in vivo*. Passive diffusion often causes premature drug leakage, inadequate therapeutic concentrations at the target site, and off-target effects, leading to a reduction in both efficacy and safety.

Stimulus-responsive strategies triggered by tumor-associated factors, such as pH-sensitive lipids that alter their structure under acidic conditions and enzyme-cleavable linkers that release payloads in response to specific proteases, remain underexplored. Incorporation of these features into biomimetic liposomes can enhance tumor-specific drug accumulation, reduce systemic exposure, and improve temporal control over therapeutic release, thereby leading to an improvement in overall treatment efficacy.

#### Production costs

The extraction of natural phospholipids and cell membrane materials, the chemical synthesis of functional lipids, and the formulation of surface modification reagents all incur high production costs. These economic barriers not only limit large-scale production but also pose challenges for commercialization and clinical translation. In addition, the complexity of purification and quality control for biologically derived components further increases production expenses and may introduce batch-to-batch variability, which complicates regulatory approval. The resolution of these cost issues is crucial to ensure both accessibility and sustainable production of biomimetic liposomes intended for repeated or high-dose clinical applications.

To address this issue, it is important to optimize the formulation by balancing the proportion of base lipids and functional additives, thereby improving cost-effectiveness. Strategies such as partial replacement of natural lipids with synthetic analogs, modular incorporation of functional components, or simplification of surface modification procedures may further reduce production costs without compromising stability or therapeutic performance. Moreover, the development of scalable and reproducible production techniques, including microfluidics and continuous extrusion systems, can enhance both efficiency and consistency, which facilitates broader clinical adoption.

### Production of biomimetic liposomes at industrial scale

The translation of biomimetic liposomes into clinical practice will depend not only on the validation of their therapeutic benefits but also on the advancement of scalable, reproducible, and economically viable manufacturing platforms that can support widespread adoption.^170^ The clinical translation of biomimetic liposomes will depend on scalable, reproducible, and cost-effective manufacturing, combined with advanced analytical platforms to ensure quality.^171^ Such developments are expected to standardize production, accelerate regulatory approval, and expand access to personalized nanomedicine.

#### Raw materials and reproducibility

The selection and standardization of raw materials are critical for large-scale production. Natural membrane sources, such as RBC membranes, immune cell membranes (*e.g.*, NK cells, macrophages), and MSC membranes, often exhibit batch-to-batch variability in lipid composition and protein content.^35,53,97^ Synthetic lipids, including DSPC, DPPC, DOPC, and PEGylated lipids, can be partially used to mitigate these variations.^170^ Standardization strategies involve using the same cell line for batch cultivation, cryopreservation or cold-chain transport to maintain membrane consistency, and modular incorporation of functional components to enhance reproducibility across batches.

#### Manufacturing techniques and scale-up

Industrial-scale production of biomimetic liposomes requires maintaining the structural integrity and biological activity of natural membrane components. Laboratory methods, such as thin-film hydration or mild sonication, allow initial formulation and membrane-lipid fusion but are unsuitable for large-volume production.^66^ Continuous extrusion and high-pressure homogenization with controlled pressure, temperature, and flow rates provide scalable methods that preserve particle uniformity and membrane protein function. Process control and post-production evaluation of membrane integrity, cellular recognition, and encapsulation efficiency ensure consistent quality and functionality, supporting reliable and scalable manufacturing for clinical applications.

#### Encapsulation and quality control

Efficient encapsulation of therapeutic agents and precise control of release profiles are critical for biomimetic liposomes, as their clinical performance depends not only on drug payload but also on the integrity and functionality of natural membrane components. For example, RBC-coated liposomes carrying doxorubicin or MSC-coated liposomes delivering nucleic acid therapeutics require careful optimization to maintain both payload retention and membrane protein activity.^53,136,163^ Comprehensive quality assessment must evaluate particle size, polydispersity, surface charge, encapsulation efficiency, chemical composition, and functional activity of membrane proteins, as well as sterility and endotoxin levels, to ensure batch-to-batch consistency. The establishment of clear batch release criteria and implementation of robust documentation procedures support regulatory approval and facilitate clinical translation of biomimetic liposomes.

#### Cost and feasibility

Economic considerations are particularly important for biomimetic liposomes, as the use of natural cell membranes and complex surface modifications can substantially increase production costs and limit widespread clinical application. Protocols that streamline production, partial replacement of natural membranes with synthetic lipids, and process optimization can reduce expenses without compromising structural integrity, membrane protein function, or therapeutic efficacy. A balance among scalability, functional performance, and cost is essential to ensure sustainable clinical translation and broader adoption of biomimetic liposomes.

## Author contributions

Zhi Li performed conceptualization, draft, and revision. Mengwen Li performed the draft. Jianqin Lu performed conceptualization, funding acquisition, and revision.

## Conflicts of interest

J.L. has applied for patents related to the Camptothesome technology. The remaining authors declare no conflicts of interest.

## Data Availability

This article does not contain any original data. All data discussed are derived from previously published studies, which are cited in the reference list.
